# A Systematic Review of Vaccination Guidance for Humanitarian Responses

**DOI:** 10.3390/vaccines11121743

**Published:** 2023-11-22

**Authors:** Lauren E. Allison, Mervat Alhaffar, Francesco Checchi, Nada Abdelmagid, Barni Nor, Majdi M. Sabahelzain, Page M. Light, Neha S. Singh

**Affiliations:** 1 Department of Infectious Disease Epidemiology and International Health, Faculty of Epidemiology and Population Health, London School of Hygiene and Tropical Medicine, London WC1E 7HT, UK; lauren.allison@lshtm.ac.uk (L.E.A.); mervat.alhaffar1@lshtm.ac.uk (M.A.); francesco.checchi@lshtm.ac.uk (F.C.); nada.abdelmagid@lshtm.ac.uk (N.A.); page.light1@alumni.lshtm.ac.uk (P.M.L.); 2 Syria Research Group, Co-Hosted between London School of Hygiene and Tropical Medicine, London, WC1E 7HT, UK and National University of Singapore Saw Swee Hock School of Public Health, Singapore 117549, Singapore; 3 Department of Women’s Children’s Health, Uppsala University, 751 05 Uppsala, Sweden; barni.nor@kbh.uu.se; 4 School of Public Health, Faculty of Medicine and Health, University of Sydney, Sydney, NSW 2006, Australia; majdisabahelzain@gmail.com; 5 Department of Global Health and Development, Faculty of Public Health and Policy, London School of Hygiene and Tropical Medicine, London WC1E 7HT, UK

**Keywords:** vaccination, guidance, humanitarian, vaccine-preventable diseases, zero dose children, crises, conflict, disaster

## Abstract

Delivering vaccines in humanitarian response requires rigourous and continuous analysis of evidence. This systematic review mapped the normative landscape of vaccination guidance on vaccine-preventable diseases in crisis-affected settings. Guidance published between 2000 and 2022 was searched for, in English and French, on websites of humanitarian actors, Google, and Bing. Peer-reviewed database searches were performed in Global Health and Embase. Reference lists of all included documents were screened. We disseminated an online survey to professionals working in vaccination delivery in humanitarian contexts. There was a total of 48 eligible guidance documents, including technical guidance (*n* = 17), descriptive guidance (*n* = 16), operational guidance (*n* = 11), evidence reviews (*n* = 3), and ethical guidance (*n* = 1). Most were World Health Organization documents (*n* = 21) targeting children under 5 years of age. Critical appraisal revealed insufficient inclusion of affected populations and limited rigour in guideline development. We found limited information on vaccines including, yellow fever, cholera, meningococcal, hepatitis A, and varicella, as well as human papilloma virus (HPV). There is a plethora of vaccination guidance for vaccine-preventable diseases in humanitarian contexts. However, gaps remain in the critical and systematic inclusion of evidence, inclusion of the concept of “zero-dose” children and affected populations, ethical guidance, and specific recommendations for HPV and non-universally recommended vaccines, which must be addressed.

## 1. Introduction

Vaccines are one of the most effective interventions to prevent morbidity and mortality [1]. Currently, the World Health Organization (WHO) recommends that all countries achieve an overarching 95% coverage threshold for all routine vaccinations to achieve herd immunity levels [1]. Despite this aim, global vaccination rates of children have declined. In 2021, the United Nations Children’s Fund (UNICEF) reported that 25 million children did not receive lifesaving vaccinations [2]. Routine vaccinations such as diphtheria–tetanus–pertussis (DTP3) also saw a decline in global coverage to 81% in 2021 from 86% in 2020 [2]. In 2020, 23 million children did not receive routine immunisation services, with up to 17 million of these children not receiving a single dose of a vaccine [3]. Zero-dose children are defined as children who have not received any routine vaccines [3]. GAVI has developed a programmatic definition for zero-dose as children who lack the first dose of diphtheria–tetanus–pertussis-containing vaccine (DTP1). Data collected prior to the coronavirus-2019 (COVID-19) pandemic identified that more than 50% of deaths from vaccine-preventable diseases (VPDs) occur among zero-dose children [4]. 

It was predicted that more than 274 million individuals would require humanitarian assistance in 2022 [5]. Crises including natural hazards such as floods or earthquakes, armed conflict, food insecurity, mass displacement, and epidemics present heightened risk for exposure to different types of VPDs. The Global Vaccine Alliance (GAVI) reports that nearly 50% of zero-dose children live in urban areas, remote communities, and in crisis-affected settings [4]. Populations in crisis-affected settings carry the double burden of a higher risk of acquiring infectious diseases and reduced access to vaccinations. This double burden underscores the need for effective strategies for delivering vaccines to crisis-affected settings, including for zero-dose children. 

Guidance documents can assist actors in responding to routine vaccination needs in crisis-affected settings using a standardised or evidence-based approach. Availability of guidance, coherence among multiple guidance sources, contextual relevance of guidance including language considerations, and credibility are identified as important considerations for guidance uptake among those responding to crisis-affected settings [6]. Several studies have investigated vaccination interventions after implementation in crisis contexts [7,8]. One scoping review assessed the availability of guidance targeting women’s, newborns’, children’s and adolescents’ health and nutrition in conflict settings and found seven documents specifically targeting immunisation [9]. Documents lacked contextualised guidance and demonstrated a limited evidence base and limited input from affected populations during their creation. A systematic review delivering infectious disease interventions to women and children in conflict settings found significant variation in practice across NGO and UN infectious disease interventions, suggesting a lack of consensus among standard operating procedures and implementation guidance [10]. To date, there has been no known review conducted to characterise the normative landscape of available guidance for vaccinating crisis-affected populations. Evaluating guideline quality and understanding the gaps and strengths of available vaccination guidance for crisis-affected settings is necessary to address gaps to support evidence-informed interventions. This review aims to systematically identify and assess available vaccination guidance for humanitarian contexts, and to identify gaps in the normative landscape for vaccination in crisis-affected settings.

## 2. Materials and Methods

### 2.1. Search Strategy 

This review followed the Preferred Reporting Items for Systematic Reviews and Meta-Analyses (PRISMA) guidelines (Table S1) [11]. The search strategy comprised five information sources, described in detail in Table S2. 

First, an anonymous survey seeking vaccination guidance was disseminated through online channels including LinkedIn, Twitter, and mailing lists between 15 July and 12 August 2022, with the aim of soliciting guidance from humanitarian health professionals involved in planning or delivering vaccination interventions in humanitarian contexts. 

Second, a manual search of grey literature published in 76 agency websites was performed. Organisations (51/76, 67%) that were members of the Global Health Cluster (GHC), and other (25/76, 33%) organisations known to be involved in humanitarian responses were selected for this manual search. For websites that contained their own search function, the key terms ‘vaccine’, ‘vaccination’, ‘humanitarian crises’, ‘conflict’, ‘disaster’, and ‘food crises’ were searched to identify relevant documents. Organisational websites that did not have a search function were manually reviewed for tools, resources, and learning sections. Titles and executive summaries of documents found in these website sections were screened for inclusion criteria. 

Third, searches using the key terms ‘guidance’, ‘guideline’, ‘vaccine’, ‘vaccination’, ‘humanitarian crises’, ‘conflict’, ‘disaster’, and ‘food crises’ were performed on Google and Bing search engines in English and French. The first 200 results of each search were screened. The first 200 results were chosen to be screened as Google and Bing search engine algorithms provide progressively less relevant results on subsequent search pages. 

Fourthly, a search strategy for peer-reviewed publications was developed using free-text search keywords including the concepts of ‘vaccination’, ‘vaccine preventable diseases’, ‘guidelines’, and ‘humanitarian emergency settings’. The first author (LA) searched the literature published in any language on Embase and Global Health between 1 January 2000 and 16 August 2022.

Finally, reference lists of documents that met inclusion criteria and the reference list of Aboubaker et al., were screened [9]. Aboubaker et al. systematically reviewed evidence on guidance for sexual, reproductive, maternal, newborn, child, and adolescent health and nutrition in conflict settings to identify gaps in guidance published between 2008 and 2018 [9]. 

### 2.2. Inclusion and Exclusion Criteria

The inclusion criteria were as follows: (1) documents published between 1 January 2000 and 16 August 2022, (2) documents included vaccination guidance for humanitarian contexts, (3) vaccination guidance focused on at least one VPD including cholera, diphtheria, hepatitis A, hepatitis B, haemophilus influenzae type b, human papilloma virus (HPV), measles, meningococcal, mumps, pertussis, polio, pneumococcal, rotavirus, rubella, tetanus, tuberculosis, varicella, and yellow fever. Twelve of these VPDs were selected for inclusion as they are universally recommended by the WHO for all routine vaccination programmes [1]. Six other non-universally WHO-recommended VPDs (yellow fever, cholera, meningococcal, mumps, hepatitis A, and varicella) were also included, as they feature an elevated risk in crisis-affected settings [12]. (4) Guidance was published in either English or French. According to the Office of the United Nations High Commissioner for Human Rights (OHCHR), a humanitarian crisis is a singular event or a series of events that threaten the safety or wellbeing of a community or large group of people [13]. Review-eligible typologies of crises included sudden mass displacement, armed conflict, food crises, natural or industrial disaster, complex humanitarian emergency, refugee camps, and epidemics. 

The following studies were excluded: (1) older versions of an updated guidance document, (2) duplicates, (3) articles for which we were unable to retrieve full texts.

All eligible records were imported into EndNote X9 for de-duplication, screening, and data extraction. Data were then extracted into Microsoft Excel 2019. 

### 2.3. Data Extraction 

Two authors (LA and NSS) independently screened titles, abstracts, or executive summaries and excluded documents that did not meet inclusion criteria. The following metavariables were extracted from the included documents: title, first author or lead development organisation, year of publication, target user, guidance type, response type, intended beneficiary group, type of humanitarian context if specified, and type(s) of VPD(s) addressed in relation to vaccination guidance. If available, additional information was extracted for each VPD including recommended vaccine dosing, age group, modality of delivery, and whether the document referred to ‘zero-dose’ children or communities. The lead organisation for the development of the document was identified by the reviewer from the first page of the document or the acknowledgements section. The target users were classified as student, journalist, immunisation programme health communications team, advocacy group, health care professional, fieldworker, international non-governmental organisations (INGOs), and immunisation programme planner, manager, or policy makers based on the intended audience stated in the document. Fieldworkers could be clinical or non-clinical staff working in crisis-affected settings, whereas healthcare professionals were clinically focused. 

The following definitions were used to classify guidance and were adapted from a similar review conducted by Aboubaker et al. [9]:

1. Technical normative guidance: defined as guidance that provides detailed information on VPD population risk factors and what to do regarding vaccination interventions. It can contain standards of care and recommendations on which vaccines to administer, age ranges, and dosing. It also includes derivative products like, for example, a routine vaccination schedule.
2. Operational guidance: defined as guidance that describes in detail how to implement interventions recommended in the technical normative guidance. It includes operational manuals, tool kits, handbooks, etc.
3. Descriptive guidance: defined as guidance that provides general information but does not include in detail what to do. This includes fact sheets, frameworks, or policy documents.
4. Ethical guidance: defined as normative guidance based on principles or conventions. The guidance may not be based on hard science or evidence but rather on a moral philosophical framework.
5. Evidence reviews: defined as a synthesis of evidence or narrative review related to a specific aspect of vaccination. Guidance is typically given as a summary of findings.

Finally, documents were classified based on the crisis phase or scenarios they focused on including acute crisis, recovery, or host countries. Full definitions for terms guiding data extraction can be found in Table S4.

### 2.4. Critical Appraisal

Due to the heterogeneity of guidance documents identified, two forms of critical appraisal tools were used. To evaluate the quality of guidelines or documents the Appraisal of Guidelines for Research and Evaluation II (AGREE II) tool was used [14]. To evaluate the quality of evidence reviews, the Scale for the Quality Assessment of Narrative Review Articles (SANRA) was used [15]. 

### 2.5. Data Analysis 

Guidelines were classified according to guidance type, target users, response type, beneficiary groups, and crisis typology. A data extraction template (Table S5), created using Microsoft Excel 2019, was used. A narrative synthesis was conducted using tabulated data to systematically compare guideline characteristics for similarities and differences between classification categories. Document quality was incorporated into the overall assessment of the strength of a document’s contribution to identified vaccine evidence. 

### 2.6. Role of Funding Source 

This work was funded by the Bill and Melinda Gates Foundation. The funder of the study had no role in the study design, data collection, data analysis, data interpretation, or writing of the report.

## 3. Results

### 3.1. Characteristics of Included Studies

**Figure 1:**
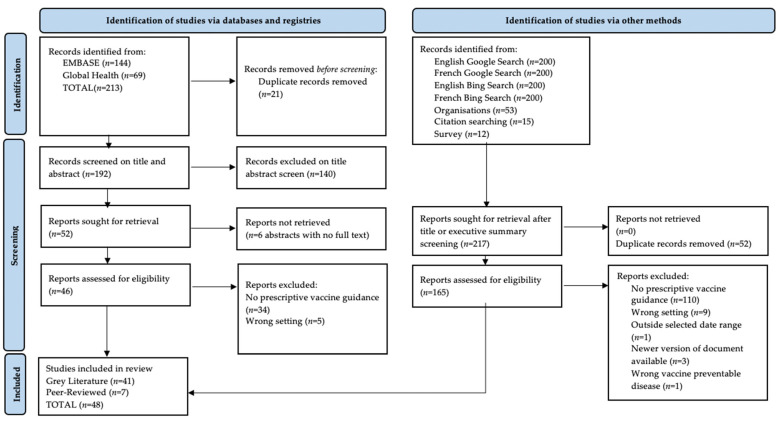
Flowchart of the vaccination guidance in humanitarian settings search according to PRISMA guidelines.

In total, 213 records were identified through peer-reviewed database searches, and an additional 880 records were identified from survey, search engine, reference lists, and manual website searches. After full-text screening, 48 documents (Figure 1) met inclusion criteria, of which 7 were peer-reviewed journal articles, and 41 were grey literature. Google and Bing search engines identified the greatest proportion of included documents (*n* = 20) followed by manual organisational website searches (*n* = 9), peer-reviewed database searches (*n* = 7), online survey (*n* = 3), and reviewing the reference list of included documents (*n* = 8) and of Aboubaker et al. (*n* = 1) (Table S3). Two documents (4%) were in French and forty-six documents (96%) in English. Documents were published between 2004 and 2022. Almost two thirds, (60%, *n* = 29) of the documents were developed in the last five years (2017–2022). The concept of zero-dose was identified in 22% (*n* = 11) of the documents, with the majority (*n* = 10) of documents identifying the concept of zero-dose being published in 2020, 2021, and 2022 (Table 1). 

**Figure 2:**
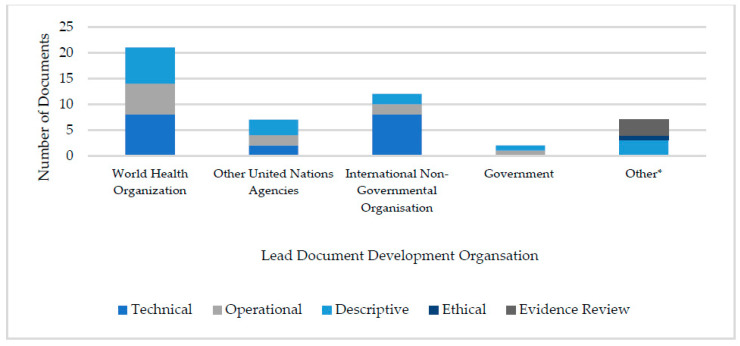
Vaccination guidance for humanitarian settings classified according to lead development organisation. * Includes documents published in academic journals such as evidence reviews.

The largest share of documents (Figure 2) were published by the WHO (*n* = 21), followed by INGOs (*n* = 12), authors in academic journals (*n* = 7), other non-WHO United Nations (UN) agencies (*n* = 6), and national governments (*n* = 2). 

### 3.2. Target Population

**Figure 3:**
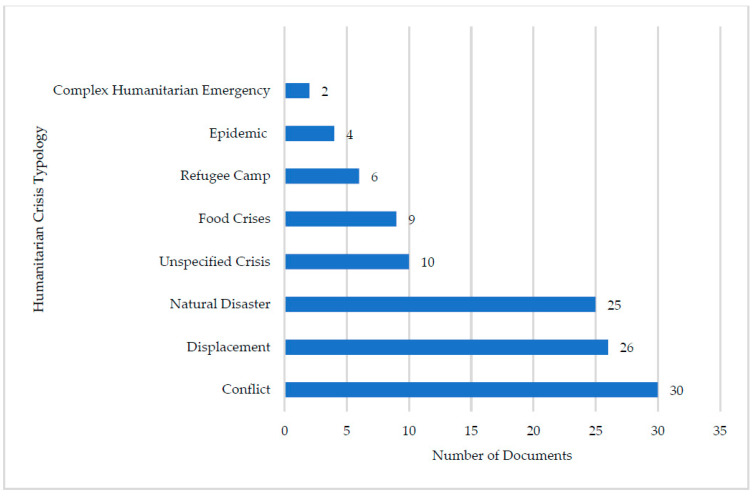
Number of vaccination guidance documents classified according to humanitarian crisis type and setting.

Nearly all guidance documents (*n* = 46, 96%) reported vaccination guidance for “children”, followed by adolescents (*n* = 18, 38%), and newborns (*n* = 2, 4%). Conflict was the most common crisis typology (*n* = 30, 63%,), followed by mass displacement (*n* = 26, 54%) and natural disasters (*n* = 25, 52%) (Figure 3). Complex humanitarian emergencies (*n* = 2, 4%) and epidemics (*n* = 4, 8%) were the least identified crises typologies. 

### 3.3. Target Users

**Figure 4:**
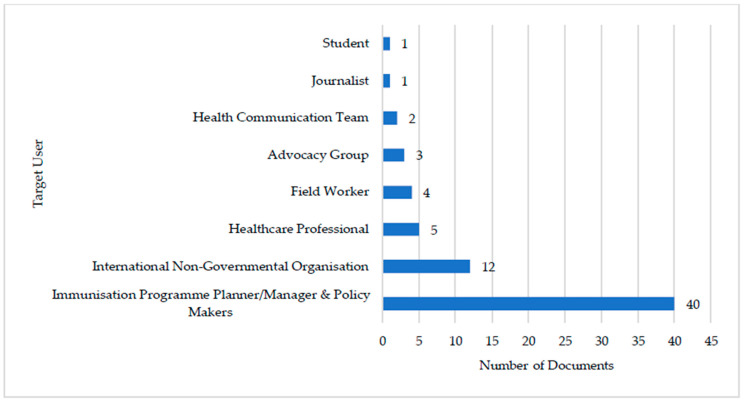
Intended target user identified by vaccination guidance document.

The majority (*n* = 40, 83%) of documents (Figure 4) were targeted towards immunization programme planners, managers, and policy makers at regional, national, or sub-national levels. Documents were targeted towards INGOs (*n* = 12, 25%), healthcare professionals (*n* = 5, 10%) and fieldworkers (*n* = 4, 8%) working in crisis-affected settings. A few documents (*n* = 3, 6%) identified guidance for use by advocacy groups and (4%, *n* = 2) health communication teams working on immunisation in crises. 

### 3.4. Vaccine-Preventable Diseases

Guidance identified in the 48 documents was mapped for each VPD (Table 1 and Table S6). Five documents (*n* = 5, 13%), all published by WHO, provided vaccination guidance for all 18 VPDs. VPDs most included in guidance (Table 2) were measles (*n* = 37, 77%), polio (*n* = 35, 73%), and tetanus (*n* = 35, 73%). VPDs that were least included were HPV (*n* = 13, 27%), as well as hepatitis A (*n* = 11, 23%) and varicella (*n* = 10, 21%) (Table 3). A total of 11 documents (23%), including for polio (*n* = 4, 8%), measles (*n* = 3, 6%), cholera (*n* = 3, 6%), and tetanus (*n* = 1, 2%), provided guidance on a single VPD. 

### 3.5. Vaccination Delivery Recommendations

Twenty-three (48%) included documents provided specific guidance related to either dosing, age groups, or modality of vaccination delivery (Table 4). Mass vaccination was the primary modality for vaccination delivery (*n* = 11, 23%), followed by strengthening of routine vaccination services (*n* = 6, 13%). Only two documents (4%) included guidance for both mass and routine modalities. All twenty-three documents with vaccination delivery recommendations provided guidance on target age group (48%). Two documents (*n* = 2, 4%) targeted catch-up vaccination and varied dosing recommendations across multiple age groups. Similarly, some guidance documents focused solely on neonatal care and did not provide vaccination dosing recommendations beyond infancy (*n* = 2, 4%). A few documents (*n* = 3, 6%) included information on target age range for vaccination, but did not indicate the recommended number of doses. One document (2%) provided contextual dosing recommendations based on time following a sexual assault for tetanus and hepatitis b.

### 3.6. Critical Appraisal 

For documents eligible for critical appraisal using the AGREE II tool (*n* = 45) (Table S7), the highest scores were in the domains of ‘Scope and Purpose’ (85%), and ‘Clarity of Presentation’ (71%). The lowest scores were in ‘Rigour of Development’ (41%) and ‘Editorial Independence’ (46%). Technical and operational guidelines scored similar, and descriptive documents scored lower overall than technical and operational documents (Table 5). Technical guidance scored higher in the domain of ‘Applicability’ (70%). The lowest scores on the 7-point Likert Scale were identified in the categories (Table S8) of ‘A procedure for updating the guideline is provided’ (1·5/7), ‘competing interests of the guideline development group members have been recorded and addressed’ (2·4/7), and ‘criteria for selecting the evidence are clearly described’ (2·4/7). Additionally, the category of “the views and preferences of the target population have been sought” was scored poorly (3·2/7) across documents. The highest scoring categories were ‘the overall objective of the guideline are specifically described’ (6·3/7), and ‘the health question(s) covered by the guideline is (are) specifically described’ (5·9/7). 

For narrative review documents that were eligible for critical appraisal (Table 6) using the SANRA tool (*n* = 3), the highest scores (Table S9) were identified in the categories of ‘Description of Literature Search’ (100%) and ‘Referencing’ (100%). The items of ‘Statement of Concrete Aims or Formulation of Questions’ (67%) and ‘Scientific Reasoning’ (67%) scored lower.

## 4. Discussion

### 4.1. Summary of Findings

To our knowledge, this is the first known review to assess the normative landscape of vaccination guidance for crisis-affected settings and to map document guidance for 18 VPDs. The review identified 48 documents that provided vaccination guidance for crisis-affected settings. There were gaps in vaccine guidance in the areas of ethics, HPV, and WHO routine vaccines not universally recommended, namely yellow fever, meningococcal, hepatitis A, mumps, and varicella. 

### 4.2. Gaps in Guidance

Since 2000, there has been an increasing availability of evidence for effective vaccine interventions for most VPDs in crisis-affected settings [7]. Despite guideline developers having a greater evidence base to inform their recommendations for vaccination, most guidance documents scored poorly in critical appraisal for the use of systematic search methods, evaluation of the current body of evidence, and reasoning for selecting evidence to inform recommendations [7]. The limited reporting of methods and reasoning for the selection of evidence could be attributable to poor methodological reporting criteria for standardised organisational guideline development processes [62]. Extensive transparency in the evidence selection assists in building credibility and trust towards guidance [6]. Credibility is identified as an important consideration in supporting the uptake of interventions for target users and beneficiary groups in crisis-affected settings [6]. 

Unsurprisingly, measles, polio, and tetanus were the most frequently identified VPDs for vaccination guidance. Polio and measles are often prioritised for mass immunisation within crisis-affected settings and require a high level of vaccine coverage to achieve herd immunity [10]. Understanding the incentives behind guidance development is also important. Global funding allocated towards different types of VPDs may be one such incentive. In recent years, there has been a greater global push to eradicate polio led by organisations such as the Global Polio Eradication Initiative [63]. Less policy and political attention from global health governance actors towards certain diseases may conversely divert funding and widen guidance gaps. 

For example, within crisis-affected settings, there is an increased prevalence of sexual violence, creating a higher exposure risk to HPV [34]. Absent or weak cervical cancer screening programmes place women at greater risk of undiagnosed cancer [34]. HPV is one of the only listed routine vaccines that protects against an illness that has a longer latency period, with onset of low-grade cervical cancer lesions five or more years after initial infection [34]. Despite decreased immediate risk of severe health outcomes in those diagnosed with HPV, ensuring the protection of girls and women through primary prevention is critical. A recent review of vaccination in crises settings found that HPV was probably not offered to affected populations in 21 of the 25 crises reviewed [32]. The lack of guidance for HPV in crisis settings may also influence how crisis responders prioritise and view the importance of providing this vaccine to crisis-affected populations. The lack of guidance for climate change-dependent VPDs such as yellow fever [7] may also be an emergent gap [64] Crisis-specific guidance on infections that have now become vaccine-preventable, including malaria and respiratory syncytial virus, may also soon become a gap. 

An important consideration is that comprehensive and detailed recommendations for one VPD may have greater usability compared to less detailed recommendations for multiple VPDs. Vaccination delivery must consider contextual factors such as limited humanitarian funding, requiring prioritisation of vaccines; unclear prior immunity levels resulting from a mixture of routine vaccination, mass campaigns, and natural exposures; potential for the integration of vaccination services with other humanitarian services such as education, child protection, and distribution of relief goods; and availability of cold chains. Furthermore, catch-up vaccination dosing schedules for specific age cohorts, how to prioritise which VPDs to address, and adapting schedules to changing VPD epidemiology require greater contextual guidance. Guidance on reporting the number of vaccinations provided in humanitarian responses was limited. This information is often required by non-governmental organisations and should be considered for inclusion in future operational guidance documents.

Included guidance in this review presented an unnecessary dichotomisation of mass and routine vaccination campaigns. There were very few guidance documents that presented both forms of vaccination campaigns or hybrid approaches. Alternative hybrid delivery modalities including leveraging other operational delivery platforms such as schools or resource distribution points should be considered [65]. Hybrid approaches should also consider the role of mobile versus static clinics as delivery modalities. Mobile clinics can assist in instances of population displacement and in the delivery of booster doses. 

Variations in dosing were commonly attributed to documents targeting different age ranges or vaccination timelines such as catch-up vaccination campaigns. Some guidance also created practical barriers for implementation. Notably, one document provided guidance, including dosing, for measles but did not contain dosing for mumps. The lack of inclusion of guidance for mumps creates added barriers for vaccination programmes intending to administer the MMR multivalent vaccine.

The term zero-dose has been more commonly adopted by INGOs such as GAVI and WHO in recent years into their vaccination strategies [49]. The novelty of the term may explain the lack of strategies targeting zero-dose children prior to 2020 from this review. 

Ethical decision making underpins a significant portion of the day-to-day work of humanitarians. Ethical challenges at the level of praxis often involve local-, cultural-, religious-, and disease-specific considerations [66]. A lack of guidance to equip humanitarians can lead to increased distress for humanitarians and affected populations, a lack of recognition of ethical dilemmas, and a lack of reflection on or assessment of the appropriate next steps. Gaps in ethical guidance at the level of humanitarian praxis in relation to vaccination are consistent with and build on findings within other areas of humanitarian response [66]. Moodley et al. have written the only guidance available to date on ethical considerations for vaccination programmes in acute humanitarian emergencies [20]. The literature assessing guidance for outbreak management in crisis-affected settings reported that ethical guidance at the level of praxis was unevenly developed to represent a range of infectious diseases and was not specific to supporting outbreak response [66]. Ethical guidance developed by the Red Cross for humanitarian professionals included guidance on assessing affected population needs and planning of interventions [67]. However, no guidance is given to assist in navigating disease or intervention-specific ethical challenges [67]. Another review highlighted a dissonance between ethical guidance documents and the problems confronting frontline humanitarian workers [68]. Moreover, consistent with this review, none of these guidance makers consulted the populations that the ethical guidance is intended for [68]. The inclusion of affected populations in guideline development can assist in supporting accessibility to mechanisms that ensure accountability of humanitarian actors. Mechanisms that promote accountability include channels to report negative reactions to humanitarian interventions, such as responses to ethical challenges or adverse medical reactions to vaccinations. This suggests a gap in the quality and availability of ethical guidance to target challenges experienced by front-line humanitarian workers and affected populations for vaccination. 

A lack of inclusion of crises-affected populations in guideline development has important implications for health communication. Affected populations can provide insights for navigating ethical challenges and supporting community acceptance of vaccination. Input from affected populations can assist in understanding and mitigating potential anti-vaccination attitudes and enable the co-development of context-specific recommendations to support informed consent. Furthermore, health communication teams were not commonly identified as target users of these guidance documents. The inclusion of health communication teams as target users and recommendations to support effective health communication is necessary to address vaccine misconceptions and support vaccine uptake in crises settings [12]. 

### 4.3. Limitations

This review has several limitations. Notably, identifying grey literature guidance documents, although carried out systematically, was limited to a manual search of organisational websites, search engines, reference lists, and a survey. Some important documents may not be publicly available and were not captured for inclusion. Similarly, search engine algorithms may have influenced the type of results produced through searches. Guidance on websites published in languages that were not English or French were also not assessed for inclusion. Moreover, the survey may not have been accessible to all key vaccination actors and may have introduced selection bias in the types of documents identified. The largest proportion of documents were published by the WHO, which may have biased the quality appraisal of the documents as the WHO has a process for guideline development that may have made the results of the data extraction and quality appraisal more homogenous [10]. Data extracted were consistent with the terminology that the guidance document identified. There may be useful guidance for other target users or intended beneficiary groups (ex: policy makers or students, etc.) within documents that were not explicitly identified by document developers.

## 5. Conclusions

Implications for policy include greater collaboration with individuals affected by humanitarian crises in guideline development and strengthening the systematic rigour of guideline development to create high-quality and evidence-based guidelines targeting contextual challenges. Individuals working at the level of humanitarian praxis, as well as those affected by crises, should be consulted in the development of ethical guidance for front-line vaccination delivery. 

Future research should build on the findings of this review and target vaccines not identified in this review such as Japanese encephalitis, tick-borne encephalitis, rabies, dengue, typhoid, and malaria [1]. These VPDs have greater disease burden within certain regions and certain high-risk populations affected by crises. Assessment of documents for guidance related to vaccine contraindications as well as how to navigate administering vaccines when information on previous vaccination is not available should also be explored. Future research should also assess characteristics of guidance documents that facilitate the usability in practice. Evaluating programme implementers’ abilities to translate evidence into practice is key to equitably and effectively meeting vaccination needs in humanitarian contexts. 

The nature of delivering routine vaccines in crisis-affected settings is complex and demands effective, evidence-based guidelines with flexible and contextual approaches which support the translation of recommendations into practice. Several vaccination guidance documents for crisis-affected settings currently exist; however, the inclusion of vaccine-specific guidance is inconsistent. Addressing gaps in guidance towards routine immunisation delivery, ethical guidance, and antigens such as HPV as well as non-universally recommended WHO routine vaccines will assist effective and evidence-informed delivery of vaccines.

## Figures and Tables

**Table 1:** Characteristics of eligible guidance documents.

No.	Lead Organisation or First Author (Year), Title	Type of Guidance	Response Type	Target User	Target Population	Humanitarian Emergency Type
1	Republic of Sudan (2005) Comprehensive Multi-Year Immunization Plan 2006–2010 [16]	Operational	Multi-response	Advocacy and communication professionals, immunisation programme managers	Infant Children Adolescent Adult	Armed conflict Natural disaster
2	Overseas Development Institute (ODI) (2007) Public Health in Crisis-Affected Populations: A Practical Guide for Decision-Makers [17]	Technical	Multi-response	Advocacy groups, Field workers, Journalists, INGOs, Policy makers, Programme officers, Students	Infant Children Pregnant women Adults	Armed conflict Entrapment Epidemic Food crises, Natural disaster Sudden unplanned displacement
3	Johns Hopkins University and Red Cross/Red Crescent (2008) Public Health Guide for Emergencies—Second Edition [18]	Technical	Multi-response	Field workers, INGOs	Neonate Children Pregnant women Adults	Armed conflict Epidemics Food crises Natural disaster Sudden unplanned displacement
4	United States Institute of Peace (2010) Defying Expectations: Polio Vaccination Programs Amid Political and Armed Conflict [19]	Descriptive	Unspecified	Immunisation programme planners	Children Adolescent Adults	Armed conflict
5	Moodley (2013) Ethical Considerations for Vaccination Programmes in Acute Humanitarian Emergencies [20]	Ethical	Acute	Immunisation programme policy makers	Children Adult	Humanitarian emergencies—unspecified
6	Lam (2015) Vaccine-Preventable Diseases in Humanitarian Emergencies Among Refugee and Internally Displaced Populations [12]	Evidence review	Multi-response	Immunisation programme planners	Children Adolescent Adult	Armed conflict Refugee camp Sudden unplanned displacement
7	Deen (2016) The Scenario Approach for Countries Considering the Addition of Oral Cholera Vaccination in Cholera Preparedness and Control Plans [21]	Descriptive	Acute	Immunisation programme planners	Children Adult	Humanitarian emergency—unspecified Natural disaster Sudden unplanned displacement
8	Finkelstein (2017) Tetanus: A Potential. Public Health Threat in Times of Disaster [22]	Evidence review	Acute	Immunisation programme planners Medical professionals	Neonate Child Adolescent Pregnant women Adult	Natural disaster
9	Nnadi (2017) Approaches to Vaccination Among Population in Areas of Conflict [23]	Descriptive	Acute	Immunisation programme planners INGOs	Children Adult	Armed conflict
10	Sphere (2018) The Sphere Handbook: Humanitarian Charter and Minimum Standards in Humanitarian Response [24]	Technical	Acute	Advocacy organisations INGOs	Infant Children Adult	Armed conflict Food crises Natural disaster
11	Jalloh (2020) Mobilize to Vaccinate: Lessons Learned from Social Mobilization for Immunization in Low- and Middle-Income Countries [25]	Descriptive	Host country	Immunisation programme planners in LMICs INGOs	Children Adolescent Adult	Humanitarian emergencies—unspecified Refugee camps
12	Save the Children (2020) Not Immune: Children in Conflict [26]	Operational	Acute	Immunisation programme planners and policy makers INGOs	Infant Children Pregnant women	Armed conflict Refugee camp Sudden unplanned displacement
13	The International Committee of the Red Cross (ICRC) (2021) ICRC Nursing Guidelines [27]	Technical	Acute	Nursing healthcare professionals	Infants Children Adults	Armed conflict Natural disaster
14	PAHO (2021) Reducing the Risk of Vaccine-Preventable Diseases in Humanitarian Emergencies [28]	Descriptive	Multi-response	Immunisation programme managers	Children Adult	Humanitarian emergencies—unspecified
15	GAVI (2021) Improving Access and Closing the Global Immunization Gap—The Critical Public Health Value of Tackling Issues of Access and Hesitancy [29]	Descriptive	Acute	Immunisation programme managers	Infant Children Adult	Armed conflict
16	GPEI (2021) Reducing Risk of Poliomyelitis Outbreaks in Emergencies [30]	Operational	Unspecified	Immunisation programme planner	Children, Adolescents Adults	Humanitarian emergencies—unspecified
17	Social Science in Humanitarian Action Platform (SSHAP) (2022) Key Considerations: Drivers Influencing Vaccination Related Behaviour Among Ukrainian Refugees in Poland [31]	Descriptive	Refugee host country	Polish immunisation programme managers	Children Adults	Sudden unplanned displacement
18	Leach (2022) The Utilisation of Vaccines in Humanitarian Crises 2015–2019: A Review of Practice [32]	Evidence review	Acute	Immunisation programme planners INGOs	Children Adolescents Adults	Armed conflict Food crises Natural disaster Sudden unplanned displacement
**Inter-Agency Working Group on Reproductive Health in Crises (IAWG)**						
19	Inter-Agency Working Group on Reproductive Health in Crises (2017) La santé du nouveau-né en situations de crise humanitaire [33]	Technical	Multi-response	Immunisation policy makers	Neonatal Infant	Armed conflict Natural disaster
20	Inter-Agency Working Group on Reproductive Health in Crises (IAWG) (2018) Inter-agency field manual on reproductive health in humanitarian settings (IAFM) [34]	Technical	Acute	Field workers	Children Adolescent Pregnant women Adult	Armed conflict Displacement Epidemics Food insecurity Natural disaster
**Médecins Sans Frontières (MSF)**						
21	MSF (2013) Management of a Measles Epidemic [35]	Technical (some operational material)	Multi-response	Immunisation programme planners Medical professionals	Children Pregnant women Adult	Armed conflict Sudden unplanned displacement
22	MSF (2018) Management of a Cholera Epidemic [36]	Technical (some operational material)	Multi-response	Immunisation programme planners, Medical professionals	Infants (>12 months) Children Adults	Armed conflict Natural disaster Sudden unplanned displacement
**United Nations High Commissioner for Refugees (UNHCR)**						
23	UNHCR (2013) Operational Guidelines on Improving Newborn Health in Refugee Operations [37]	Operational	Acute	Immunisation programme planner	Newborn	Displacement Refugee camp
24	UNHCR (2015) UNHCR Emergency Handbook [13]	Technical	Acute	Immunisation programme planners and management INGOs	Infant Children Adolescents	Mass displacement Refugee camps
**United Nations Children’s Fund (UNICEF)**						
25	UNICEF (2004) Reducing Measles Mortality in Emergencies [38]	Descriptive	Unspecified	Immunisation programme planners	Children Adults	Humanitarian emergencies—unspecified
26	UNICEF (2005) Emergency Field Handbook [39]	Technical	Acute	Field workers	Children Adult	Armed conflict Displacement Natural disaster
27	UNICEF, Public Good Project (PGP), Yale Institute for Global Health (2020) Vaccine Misinformation Field Guide [40]	Operational	Unspecified	Health communication teams Immunisation programme planners	Children Adults	Armed conflict Natural disaster Outbreak
28	UNICEF (2021) Lessons Learned and Practices: Country-Specific Case Studies on Immunization Activities During the COVID-19 Pandemic [41]	Descriptive	Acute	Immunisation programme planners	Neonate Infant Children Adolescent Pregnant women Adult	Armed conflict Food insecurity Natural disaster Sudden unplanned displacement
**World Health Organization (WHO)**						
29	WHO (2005) Communicable Disease Control in Emergencies [42]	Operational	Unspecified	Immunisation programme planners	Children Adolescent Adult	Sudden unplanned displacement Refugee camp Natural disaster Food crises
30	WHO (2006) Communicable Diseases Following Natural Disasters: Risk Assessment and Priority Interventions [43]	Technical	Acute	INGOs	Children Adolescents Pregnant women Adults	Natural disasters Outbreaks
31	WHO (2008) Manual for the Healthcare of Children in Humanitarian Emergencies [44]	Technical	Multi-response	Medical professionals	Neonate Children—under 5 years old	Humanitarian emergency—unspecified
32	WHO (2010) Oral Cholera Vaccine in Mass Immunization Campaigns [45]	Technical	Unspecified	Immunisation programme planners INGOs	Children, Adolescent Adult	Armed conflict Epidemic Natural disaster Outbreak Sudden unplanned displacement
33	WHO—UNHCR—UNICEF (2015) Joint Statement on General Principles on Vaccination of Refugees, Asylum-Seekers, and Migrants in the WHO European Region [46]	Descriptive	Unspecified	Immunisation programme planners	Children Adolescent Adults	Sudden unplanned displacement
34	WHO (2016) Planning and Implementing High-Quality Supplementary Immunization Activities for Injectable Vaccines Using an Example of Measles and Rubella Vaccines [47]	Operational	Acute	Immunisation programme managers	Infant Children Pregnant women Adult	Armed conflict Sudden unplanned displacement
35	WHO (2016) Global Routine Immunization on Strategies and Practices [48]	Descriptive	Multi-response	Immunisation programme planners INGOs	Neonate Infant Children Adolescent Pregnant women Adult	Armed conflict Humanitarian emergencies—unspecified
36	WHO (2017) Vaccination in Acute Humanitarian Emergencies: A Framework for Decision Making [49]	Technical	Acute	Senior-level government programme planner Partner INGO	Neonates Children Adolescents Pregnant women Adults	Armed conflict Breakdown of critical administrative and management functions Food crises Natural or industrial disaster Sudden unplanned displacement
37	WHO (2017) Vaccination in Acute Humanitarian Emergencies: Implementation Guide [50]	Operational	Acute	Programme planner and managers	Neonates Children Adolescents Pregnant women Adults	Armed conflict Breakdown of critical administrative and management functions Food crises Natural or industrial disaster Sudden unplanned displacement
38	WHO (2019) Polio Endgame Strategy 2019–2023: Eradication, Integration, Certification and Containment [51]	Descriptive	Multi-response	INGOs Immunisation programme planners	Infants Children Adults	Armed conflict Humanitarian emergencies—unspecified
39	WHO (2020) Delivery of Immunization Services for Refugees and Migrants—Technical Guidance [52]	Technical	Host country	Policy makers and immunisation programme planners	Infants Children Adolescents Pregnant women Adults	Sudden unplanned displacement
40	WHO (2020) Procédures opérationnelles standardisées riposte à un évènement ou à une flambée de poliomyélite [53]	Operational	Unspecified	Immunisation programme managers	Children Adult	Armed conflict Natural disaster Sudden unplanned displacement
41	WHO (2020) Making a Comprehensive Annual National Immunization [54]	Operational	Unspecified	Mid-level managers at national or provincial level	Infant Children Adult	Armed conflict
42	WHO (2020) Immunization as an Essential Health Service: Guiding Principles for Immunization Activities during the COVID-19 Pandemic and other Times of Severe Disruption [55]	Descriptive	Catch-up	Immunisation programme planners	Children Adult	Humanitarian emergencies—unspecified Natural disasters
43	WHO (2021) Leave No One Behind—Guidance for Planning and Implementing Catch-up Vaccination [56]	Technical	Catch-up	Immunisation programme planners	Neonates Children Adolescents Adults	Armed conflict epidemic Natural disaster outbreak Sudden unplanned displacement
44	WHO (2021) Immunization Agenda 2030: Coverage and Equity [57]	Descriptive	Acute	Immunisation programme managers	Neonatal Children Adolescent Adult	Armed conflict Sudden unplanned displacement
45	WHO (2022) Ensuring the Integration of Refugees and Migrants in Immunization Policies, Planning, and Service Delivery Globally [58]	Operational	Multi-response	Immunisation programme planner and policy makers	Children Adolescents Adults	Armed conflict Natural disasters Refugee camps Sudden unplanned displacement
46	WHO (2022) Guidance on Vaccination and Prevention of Vaccine-Preventable Disease Outbreaks for Countries Hosting Refugees from Ukraine [59]	Technical	Host Country	Immunisation programme planners	Children Adolescent Adult	Sudden unplanned displacement
47	WHO (2022) Regional Strategic Framework for Vaccine-Preventable Diseases and Immunization in the Western Pacific 2021–2030 [60]	Descriptive	Multi-response	Immunisation programme planners INGOs	Neonate Infant Children Adolescent Pregnant women Adult	Natural disasters Outbreaks Sudden unplanned displacement
48	WHO (2022) Guiding Principles for Recovering, Building Resiliency, and Strengthening of Immunization in 2022 and Beyond [61]	Descriptive	Catch-up	Immunisation programme planners	Children Adolescents Pregnant women Adult	Armed conflict Natural disaster Outbreak

**Table 2:** Percentage of vaccine guidance for VPD mapped to corresponding WHO routine vaccines recommended for all immunisation programmes (1).

WHO Vaccines Recommended for All Immunization Programs	Percentage of Documents Containing Vaccine Guidance for Corresponding Vaccine-Preventable Disease (*n*/48 Guidance Documents)
BCG (Bacillus Calmette–Guérin)	40% (19/48)
Hepatitis B	56% (27/48)
Polio	73% (35/48)
DTP (Diphtheria, Tetanus, Pertussis)	50% (24/48), 67% (32/48), 48% (23/48)
Hib (Haemophilus influenzae type b)	38% (18/48)
Pneumococcal	38% (18/48)
Rotavirus	31% (15/48)
Measles	77% (37/48)
Rubella	44% (21/48)
HPV	27% (13/48)

**Table 3:** Percentage of other vaccines assessed in review mapped according to WHO non-universal routine vaccine programme recommendations (1).

WHO Non-Universal Routine Vaccines Recommended	Percentage of Documents Containing Vaccine Guidance for Corresponding Vaccine-Preventable Disease (*n*/48 Guidance Documents)
**Vaccines recommended for certain regions**	
Yellow Fever	40% (19/48)
**Vaccines recommended for some high-risk populations**	
Cholera	50% (24/48)
Meningococcal	31% (15/48)
Hepatitis A	23% (11/48)
**Recommendations for immunisation programs with certain characteristics**	
Mumps	33% (16/48)
Varicella	21% (10/48)

**Table 4:** Vaccination recommendations.

Vaccine	Document Name	Recommended Dose		Target Age Group		Modality
**BCG**	Vaccination in Acute Humanitarian Emergencies—A framework for decision making	1 dose		Neonates		Unspecified
	WHO—Leave No One Behind—Guidance for Planning and Implementing Catch-up Vaccination	1 dose		Neonates/Birth		Unspecified
		1 dose		≤12 months		Catch-up vaccination (mass and routine)
		1 dose		>12 months		
	Manual for the Healthcare of Children in Humanitarian Emergencies	1 dose		Birth		Unspecified
	Guidance on Vaccination and Prevention of Vaccine-Preventable Disease Outbreaks for Countries Hosting Refugees from Ukraine	1 dose		3–5 days old		Routine vaccination
	Comprehensive Multi-Year Immunization Plan 2006–2010	1 dose		Birth		Routine vaccination
	La santé du nouveau-né en situations de crise humanitaire	Unspecified		Birth		Routine vaccination
	Regional Strategic Framework for Vaccine-Preventable Diseases and Immunization in the Western Pacific 2021–2030	1 dose		Birth dose—within 24 h		Routine and mass vaccination
	Communicable Disease Control in Emergencies	1 dose		Newborns		Routine vaccination
**Hepatitis B**	Vaccination in Acute Humanitarian Emergencies—A framework for decision making	3 doses		Birth dose—within 24 h		Unspecified
	WHO—Leave No One Behind—Guidance for Planning and Implementing Catch-up Vaccination	3–4 doses		Birth dose—within 24 h 6 weeks, 10 weeks, 14 weeks (4-week interval between doses)		Routine and mass vaccination
		3 doses		≤12 months		Catch-up vaccination (mass and routine)
		3 doses		>12 months		
	Manual for the Healthcare of Children in Humanitarian Emergencies	3 doses		6 weeks, 10 weeks, 14 weeks		Unspecified
	Guidance on Vaccination and Prevention of Vaccine-Preventable Disease Outbreaks for Countries Hosting Refugees from Ukraine	3 doses		Birth, 2 months, 6 months		Routine vaccination
	Comprehensive Multi-Year Immunization Plan 2006–2010	3 doses		6 weeks, 10 weeks, 14 weeks		Routine vaccination
	La santé du nouveau-né en situations de crise humanitaire	Unspecified		Birth		Routine vaccination
	Inter-agency field manual on reproductive health in humanitarian setting	3 doses		14 days after sexual assault, 4 weeks after first dose, 8 weeks after second dose		Routine vaccination
	Regional Strategic Framework for Vaccine-Preventable Diseases and Immunization in the Western Pacific 2021–2030	2 doses		Birth dose—within 24 h, within 1 year		Routine and mass vaccination
	Operational Guidelines on Improving Newborn Health in Refugee Operations	1 dose (first dose of series)		Birth dose—within 24 h		Routine vaccination
**Polio** **bOPV:** Bivalent Oral Polio Vaccine **IPV:** Inactivated Poliovirus Vaccine	Vaccination in Acute Humanitarian Emergencies—A framework for decision making	3 doses (bOPV) 1 dose (IPV)		Birth dose (first bOPV) ≥14 weeks (IPV)		Unspecified
	WHO—Leave No One Behind—Guidance for Planning and Implementing Catch-up Vaccination	4 doses (bOPV + IPV)		6 weeks (first bOPV) ≥14 weeks (IPV)		Catch-up vaccination (mass and routine)
		4 doses (1–2 IPV and 2 OPV Sequential)		8 weeks (first IPV—4 weeks minimum interval between subsequent doses)		
		3 doses (IPV)		8 weeks (4 weeks minimum interval between subsequent doses)		
	Manual for the Healthcare of Children in Humanitarian Emergencies	4 doses		Birth, 6 weeks, 10 weeks, 14 weeks		Unspecified
	Guidance on Vaccination and Prevention of Vaccine-Preventable Disease Outbreaks for Countries Hosting Refugees from Ukraine	2 doses (IPV) 4 doses (OPV)		2 months, 4 months 6 months, 18 months, 6 years, 14 years		Routine vaccination
	Comprehensive Multi-Year Immunization Plan 2006–2010	3 doses		6 weeks, 10 weeks, 14 weeks		Routine vaccination
	La santé du nouveau-né en situations de crise humanitaire	1 dose		Birth dose		Routine vaccination
	Procédures opérationnelles standardisées riposte à un évènement ou à une flambée de poliomyélite	2 doses		Under age of 5 years		Mass vaccination
	Reducing the Risk of Vaccine-Preventable Diseases in Humanitarian Emergencies	3 doses		Under age of 5 years up to 15 years		Mass vaccination
	Regional Strategic Framework for Vaccine-Preventable Diseases and Immunization in the Western Pacific 2021–2030	Unspecified		<1 year old		Routine and mass vaccination
	Operational Guidelines on Improving Newborn Health in Refugee Operations	1 dose (separate from primary series)		Birth dose (does not count towards primary dosing schedule)		Routine vaccination
	Reducing Risk of Poliomyelitis Outbreaks in Emergencies	3 doses		<5 years (3–4 weeks intervals between doses, shorter intervals if high risk population)		Mass vaccination
**Diphtheria, Tetanus, and Pertussis** **TT:** Tetanus Toxoid **DT:** Diphtheria Tetanus **Td:** Tetanus, Diphtheria **DTP-Hep B-Hib:** Diphtheria, Tetanus, Pertussis, Hepatitis B, Haemophilus Influenzae Type B	Vaccination in Acute Humanitarian Emergencies—A framework for decision making	5 doses (TT)		Infancy, adult		Unspecified
		5 doses (DT)		Infancy, <7 years old		
		3 doses (Td)		≥7 years old, adults		
		3 doses (DTP-Hep B- Hib pentavalent)		≥6 weeks to <7 years old		
		3 doses (DTP)		≥6 weeks to <7 years, pregnant women		
		3 doses (DTP—Hib)		≥6 weeks to <2 years		
	WHO—Leave No One Behind—Guidance for Planning and Implementing Catch-up Vaccination	3 doses (DTP)	+ 3 booster doses	6 weeks (4 weeks minimum interval between subsequent doses)	Booster: 12–23 months (DTP-containing vaccine); 4–7 years (Td/DT containing vaccine); and 9–15 years (Td containing vaccine)	Routine and mass vaccination
		3 doses (DTP)		≤12 months of age		Catch-up vaccination (Mass and Routine)
		3 doses (DTP)		>12 months of age (4 weeks interval between 1st and 2nd dose, 6-month interval between 2nd and 3rd dose)		
	Public Health Guide for Emergencies—Second Edition	2 doses (TT)		All children		Mass vaccination
		2 doses (Td)		All children		
	Manual for the Healthcare of Children in Humanitarian Emergencies	3 doses (DPT-Hib)		6 weeks, 10 weeks, 14 weeks		Unspecified
	Guidance on Vaccination and Prevention of Vaccine-Preventable Disease Outbreaks for Countries Hosting Refugees from Ukraine	6 doses (DT)		2 months, 4 months, 6 months, 18 months, 6 years, 16 years, booster every 10 years		Routine vaccination
		4 doses (pertussis)		2 months, 4 months, 6 months, 18 months		
	ICRC Nursing Guidelines	5 doses (tetanus—TT or Td)		Arrival to crisis setting, 4 weeks after first dose, 6 months–1 year after second dose or following pregnancy, 1–5 years after third dose or following pregnancy, 1–10 years after fourth dose or following pregnancy		Unspecified
	Comprehensive Multi-Year Immunization Plan 2006–2010	3 doses (DPT)		6 weeks, 10 weeks, 14 weeks		Routine vaccination
		5 doses (tetanus)		First contact, one month after dose 1, 6 months after dose 2, 1 year after dose 3, 1 year after dose 4		
	La santé du nouveau-né en situations de crise humanitaire	2 doses (tetanus)		Pregnant women		Routine vaccination
	Inter-agency field manual on reproductive health in humanitarian setting	3 doses (tetanus)		After assault, 4 weeks after dose 1, 6 months to 1 year after dose 2		Routine vaccination
	Regional Strategic Framework for Vaccine-Preventable Diseases and Immunization in the Western Pacific 2021–2030	4 doses (Td/Tdap)		<1 year, 1–9 years, 10–19 years, 20–65 years		Routine and mass vaccination
	Tetanus: A Potential. Public Health Threat in Times of Disaster	5 doses (Dtap)		2 months, 4 months, 6 months, 12–15 months, 4–6 years		Unspecified
	UNICEF Emergency Field Handbook	5 doses (tetanus)		Childbearing age 15–49, 4 weeks after dose 1, 6 months after dose 2, 1 year after dose 3, 1 year after dose 4		Mass vaccination
	Operational Guidelines on Improving Newborn Health in Refugee Operations	2 doses (tetanus)		Antenatal pregnant women (at least 4 weeks apart)		Routine Vaccination
	Communicable Disease Control in Emergencies	3 doses (DTP-TT)		All children aged 0–1 year		Mass vaccination
**Hib**	Guidance on Vaccination and Prevention of Vaccine-Preventable Disease Outbreaks for Countries Hosting Refugees from Ukraine	3 doses (Hib)		2 months, 4 months, 12 months		Routine vaccination
**Pneumococcal** **DTPCV:** Diphtheria, Tetanus, Pertussis containing vaccine **PCV:** Pneumococcal Conjugate Vaccine	Vaccination in Acute Humanitarian Emergencies—A framework for decision making	3 doses		6 weeks to 5 years		Unspecified
	WHO—Leave No One Behind—Guidance for Planning and Implementing Catch-up Vaccination	3 doses (with DTPCV)		6 weeks (4-week intervals between dosing)		Routine and mass vaccination
		2 doses (PCV)		6 weeks (8 weeks intervals between dosing)		
		2–3 doses		≤12 months		Catch-up vaccination (mass and routine)
		2 doses		>12 months (1–5 years at high-risk)		
	Regional Strategic Framework for Vaccine-Preventable Diseases and Immunization in the Western Pacific 2021–2030	Unspecified		<1 year		Routine and mass vaccination
**Rotavirus**	Vaccination in Acute Humanitarian Emergencies—A framework for decision making	3 doses (RotaTeq Liquid)		6 weeks to 2 years		Unspecified
		2 doses (Rotavirus)		6 weeks to 2 years		
		2 doses (Rotarix liquid)		6 weeks to 2 years		
	WHO—Leave No One Behind—Guidance for Planning and Implementing Catch-up Vaccination	2 or 3 doses		6 weeks		Routine and mass vaccination
		2 or 3 doses		≤12 months of age		Catch-up vaccination (mass and routine)
		2 or 3 doses		>12 months of age (limited benefit for those >24 months)		
	ICRC Nursing Guidelines	Unspecified		6 weeks to 24 months		Unspecified
	Regional Strategic Framework for Vaccine-Preventable Diseases and Immunization in the Western Pacific 2021–2030	Unspecified		<1 year		Routine and mass vaccination
**Measles** **MR:** Measles, Rubella **MMR:** Measles, Mumps, Rubella **MCV:** Measles Containing Vaccine **MRCV:** Measles Rubella Containing Vaccine **MMRV:** Measles, Mumps, Rubella, Varicella	Vaccination in Acute Humanitarian Emergencies—A framework for decision making	2 doses (Measles)		≥9 months (≥6 months in outbreak settings)		Unspecified
		2 doses (MR)		≥9 months (≥6 months in outbreak settings)		
		2 doses (MMR)		≥9 months (≥6 months in outbreak settings)		
	WHO—Leave No One Behind—Guidance for Planning and Implementing Catch-up Vaccination	2 doses (MCV)		9 or 12 months (minimum 4-week intervals between doses)		Routine and mass vaccination
		2 doses (MCV)		≤12 months		Catch-up vaccination (mass and routine)
		2 doses (MCV)		>12 months		
	Manual for the Healthcare of Children in Humanitarian Emergencies	2 doses		9 months (minimum age of 6–9 months, with revaccination at 9 months)		Unspecified
	Guidance on Vaccination and Prevention of Vaccine-Preventable Disease Outbreaks for Countries Hosting Refugees from Ukraine	2 doses (MRCV)		Up to 15 years		Routine vaccination
		2 doses (MMR)		12 months, 6 years		
	MSF—Management of a Measles Epidemic	2 doses (MR/MMR/MMRV)		High transmission: 9 months Low transmission: 12–15 months 2nd dose between 6 months and 5 years old		Mass vaccination
	Comprehensive Multi-Year Immunization Plan 2006–2010	1 dose		9 months		Routine vaccination
	UNHCR Emergency Handbook	2 doses		6 months—(need to repeat vaccine over 9 months of age, up to 15 years old)		Mass vaccination
	Not Immune: Children in Conflict	2 doses		9–12 months, 15–18 months		Mass vaccination
	Planning and Implementing High-Quality Supplementary Immunization Activities for Injectable Vaccines Using an Example of Measles and Rubella Vaccines	3 doses		High Transmission: 6 months Low Transmission: 9–12 months Second vaccine at least 1 month after first		Mass vaccination
	Reducing the Risk of Vaccine-Preventable Diseases in Humanitarian Emergencies	Unspecified		<5 years old		Mass vaccination
	Regional Strategic Framework for Vaccine-Preventable Diseases and Immunization in the Western Pacific 2021–2030	Unspecified		<1 year, 1–9 years		Routine and mass vaccination
	UNICEF Emergency Field Handbook	2 doses		6 months–14 years, Children under 9 months must receive a second dose of measles vaccine at 9 months of age but not less than 30 days after dose 1		Mass vaccination
	Communicable Disease Control in Emergencies	2 doses		First dose at 6–8 months, second dose at 9 months		Mass vaccination
**Rubella**	WHO—Leave No One Behind—Guidance for Planning and Implementing Catch-up Vaccination	1 dose		9 or 12 months		Routine and mass vaccination
		1 dose		≤12 months		Catch-up vaccination (mass and routine)
		1 dose		>12 months		
	Regional Strategic Framework for Vaccine-Preventable Diseases and Immunization in the Western Pacific 2021–2030	Unspecified		<1 year		Routine and mass vaccination
**HPV**	Vaccination in Acute Humanitarian Emergencies—A framework for decision making	2 doses (Cervarix or Gardasil)		9–13 years		Unspecified
	WHO—Leave No One Behind—Guidance for Planning and Implementing Catch-up Vaccination	2 doses (if started <15 years, 5 months between doses) or 3 doses (if started ≥15 years, 1-month interval for 1st dose, 4-months interval for 3rd dose)		≥9 years old		Catch-up vaccination (mass and routine)
	Inter-agency field manual on reproductive health in humanitarian setting	3 doses		≤26 years old (give 3 doses over 6 months)		Routine vaccination
	Regional Strategic Framework for Vaccine-Preventable Diseases and Immunization in the Western Pacific 2021–2030	Unspecified		10–19 years		Routine and mass vaccination
**Yellow Fever**	Vaccination in Acute Humanitarian Emergencies—A framework for decision making	1 dose		≥9 months		Unspecified
	Communicable Disease Control in Emergencies	1 dose		≥6 months		Mass vaccination
**Cholera** **OCV:** Oral Cholera Vaccine	Vaccination in Acute Humanitarian Emergencies—A framework for decision making	2–3 doses (Dukoral)		≥2 years		Unspecified
		2 doses (Shanchol/Euvichol)		≥1 year		
	Management of a Cholera Epidemic	2–3 doses (Dukoral—age dependent)		<6 years old (3 doses) ≥6 years old (2 doses)		Mass vaccination
		2 doses (Shanchol/Euvichol)		≥12 months (14 days between doses)		
	Oral Cholera Vaccine in Mass Immunization Campaigns	2 doses (OCV)		≥2 years old		Mass vaccination
**Meningococcal** **Men A/C:** Meningococcal A/C	Vaccination in Acute Humanitarian Emergencies—A framework for decision making	1 dose (Men A/C/MenAfriVacA)		1–29 years		Unspecified
	Regional Strategic Framework for Vaccine-Preventable Diseases and Immunization in the Western Pacific 2021–2030	Unspecified		<1 year, 1–9 years, 10–19 years		Routine and mass vaccination
	Communicable Disease Control in Emergencies	1 dose		2–10 years		Mass vaccination
**Hepatitis A**	Vaccination in Acute Humanitarian Emergencies—A framework for decision making	1 dose		≥1 year		Unspecified
	Regional Strategic Framework for Vaccine-Preventable Diseases and Immunization in the Western Pacific 2021–2030	Unspecified		1–9 years, 10–19 years		Routine and mass vaccination
**Mumps** **MMR:** Measles, Mumps, and Rubella	Vaccination in Acute Humanitarian Emergencies—A framework for decision making	2 doses (MMR)		≥9 months (can be considered in high-risk settings ≥6 months)		Unspecified
	Guidance on Vaccination and Prevention of Vaccine-Preventable Disease Outbreaks for Countries Hosting Refugees from Ukraine	2 doses (MMR)		12 months, 6 years		Routine vaccination
	Regional Strategic Framework for Vaccine-Preventable Diseases and Immunization in the Western Pacific 2021–2030	Unspecified		<1 year		Routine and mass vaccination
**Varicella** **MMRV:** Measles, Mumps, Rubella, and Varicella	Vaccination in Acute Humanitarian Emergencies—A framework for decision making	1 or 2 doses (Varicella or MMRV)		≥9 months		Unspecified
	Regional Strategic Framework for Vaccine-Preventable Diseases and Immunization in the Western Pacific 2021–2030	Unspecified		<1 year		Routine and mass vaccination

**Table 5:** AGREE II quality appraisal scores by guidance type.

Document Type	Number of Documents Eligible for AGREE II	Mean AGREE II Scores for Six Domains					
		Scope and Purpose	Stakeholder Involvement	Rigour of Development	Clarity of Presentation	Applicability	Editorial Independence
Technical	17	91%	60%	44%	76%	70%	47%
Operational	11	84%	60%	44%	71%	57%	43%
Descriptive	16	78%	50%	38%	65%	47%	48%
Ethical	1	81%	67%	30%	67%	36%	36%
**Total**	**45**	**85%**	**57%**	**41%**	**71%**	**58%**	**46%**

**Table 6:** SANRA quality appraisal scores for evidence reviews.

Document Type	Number of Documents Eligible for SANRA	Mean SANRA Scores for Six Items					
		Justification of the Article’s Importance for the Readership	Statement of Concrete Aims or Formulation of Questions	Description of Literature Search	Referencing	Scientific Reasoning	Appropriate Presentation of Data
Evidence reviews	3	83%	67%	100%	100%	67%	83%

## Data Availability

The data presented in this study are available in the supplementary materials.

## Supplementary Materials

The following supporting information can be downloaded at: https://www.mdpi.com/article/10.3390/vaccines11121743/s1, Table S1: PRISMA Checklist; Table S2: Search strategy; Table S3: List of studies included in the review; Table S4: Data extraction classification definitions; Table S5: Data extraction framework; Table S6: Vaccine-preventable diseases included in guidance documents; Table S7: AGREE II critical appraisal by domain; Table S8: AGREE II Critical appraisal disaggregated by individual domain indicators; Table S9: SANRA Critical appraisal by item.
