# Supplementary material for: A Systematic Review of Vaccination Guidance for Humanitarian Responses
Source: Vaccines (Basel). 2023 Nov 22;11(12):1743. doi: 10.3390/vaccines11121743 (PMC10747938; doi:10.3390/vaccines11121743)
Supplement: Supplementary file 1 [file vaccines-11-01743-s001.zip › vaccines-2701516-supplementary.pdf]

SUPPLEMENTARY FILE

CONTENTS

Supplementary Tables

Table S1. PRISMA Checklist……………………………………………………………………….………2
Table S2. Search Strategy……………………………..…………………………………………...….….…..6
Table S3. List of studies included in the review……………………………………………………………14
Table S4. Data extraction and classification definitions..……………………...…………………….……18
Table S5. Data extraction framework...…………………………………………………………………..…19
Table S6. Vaccine preventable diseases included in the guidance documents………………………….…21
Table S7. AGREE II Critical appraisal by domain...……………………………………………………..…22
Table S8. AGREE II Critical appraisal disaggregated by individual domain indicators………...….……26
Table S9. SANRA Critical appraisal by item……………………………………………………….………27

SUPPLEMENTARY TABLES

Table S1. Preferred Reporting Items for Systematic Reviews and Meta-analyses (PRISMA) Checklist 2020

Section and Topic	Item #	Checklist item	Location where item is reported
**TITLE**			
Title	1	Identify the report as a systematic review.	Page 1
**ABSTRACT**			
Abstract	2	See the PRISMA 2020 for Abstracts checklist.	Page 1
**INTRODUCTION**			
Rationale	3	Describe the rationale for the review in the context of existing knowledge.	Page 2
Objectives	4	Provide an explicit statement of the objective(s) or question(s) the review addresses.	Page 2
**METHODS**			
Eligibility criteria	5	Specify the inclusion and exclusion criteria for the review and how studies were grouped for the syntheses.	Page 3
Information sources	6	Specify all databases, registers, websites, organisations, reference lists and other sources searched or consulted to identify studies. Specify the date when each source was last searched or consulted.	Page 2-3 & Tables S2 and S3
Search strategy	7	Present the full search strategies for all databases, registers and websites, including any filters and limits used.	Page 2 & Table S2
Selection process	8	Specify the methods used to decide whether a study met the inclusion criteria of the review, including how many reviewers screened each record and each report retrieved, whether they worked independently, and if applicable, details of automation tools used in the process.	Page 3
Data collection process	9	Specify the methods used to collect data from reports, including how many reviewers collected data from each report, whether they worked independently, any processes for obtaining or confirming data from study investigators, and if applicable, details of automation tools used in the process.	Page 3-4
Data items	10a	List and define all outcomes for which data were sought. Specify whether all results that were compatible with each outcome domain in each study were sought (e.g. for all measures, time points, analyses), and if not, the methods used to decide which results to collect.	Page 3-4
	10b	List and define all other variables for which data were sought (e.g. participant and intervention characteristics, funding sources). Describe any assumptions made about any missing or unclear information.	Page 4

Study risk of bias assessment	11	Specify the methods used to assess risk of bias in the included studies, including details of the tool(s) used, how many reviewers assessed each study and whether they worked independently, and if applicable, details of automation tools used in the process.	Page 4
Effect measures	12	Specify for each outcome the effect measure(s) (e.g. risk ratio, mean difference) used in the synthesis or presentation of results.	Not applicable
Synthesis methods	13a	Describe the processes used to decide which studies were eligible for each synthesis (e.g. tabulating the study intervention characteristics and comparing against the planned groups for each synthesis (item #5)).	Page 4
	13b	Describe any methods required to prepare the data for presentation or synthesis, such as handling of missing summary statistics, or data conversions.	Not applicable
	13c	Describe any methods used to tabulate or visually display results of individual studies and syntheses.	Page 4
	13d	Describe any methods used to synthesize results and provide a rationale for the choice(s). If meta-analysis was performed, describe the model(s), method(s) to identify the presence and extent of statistical heterogeneity, and software package(s) used.	Page 4
	13e	Describe any methods used to explore possible causes of heterogeneity among study results (e.g. subgroup analysis, meta-regression).	Not applicable
	13f	Describe any sensitivity analyses conducted to assess robustness of the synthesized results.	Not applicable
Reporting bias assessment	14	Describe any methods used to assess risk of bias due to missing results in a synthesis (arising from reporting biases).	Not applicable
Certainty assessment	15	Describe any methods used to assess certainty (or confidence) in the body of evidence for an outcome.	Not applicable

Section and Topic	Item #	Checklist item	Location where item is reported
**RESULTS**			
Study selection	16a	Describe the results of the search and selection process, from the number of records identified in the search to the number of studies included in the review, ideally using a flow diagram.	Page 6
	16b	Cite studies that might appear to meet the inclusion criteria, but which were excluded, and explain why they were excluded.	Page 6, Figure 1
Study characteristics	17	Cite each included study and present its characteristics.	Page 5

Risk of bias in studies	18	Present assessments of risk of bias for each included study.	Table S4 and Table S9
Results of individual studies	19	For all outcomes, present, for each study: (a) summary statistics for each group (where appropriate) and (b) an effect estimate and its precision (e.g. confidence/credible interval), ideally using structured tables or plots.	Not applicable
Results of syntheses	20a	For each synthesis, briefly summarise the characteristics and risk of bias among contributing studies.	Table S7, and Table S9
	20b	Present results of all statistical syntheses conducted. If meta-analysis was done, present for each the summary estimate and its precision (e.g. confidence/credible interval) and measures of statistical heterogeneity. If comparing groups, describe the direction of the effect.	Not applicable
	20c	Present results of all investigations of possible causes of heterogeneity among study results.	Not applicable
	20d	Present results of all sensitivity analyses conducted to assess the robustness of the synthesized results.	Not applicable
Reporting biases	21	Present assessments of risk of bias due to missing results (arising from reporting biases) for each synthesis assessed.	Not applicable
Certainty of evidence	22	Present assessments of certainty (or confidence) in the body of evidence for each outcome assessed.	Not applicable
**DISCUSSION**			
Discussion	23a	Provide a general interpretation of the results in the context of other evidence.	Page 26
	23b	Discuss any limitations of the evidence included in the review.	Page 27
	23c	Discuss any limitations of the review processes used.	Page 27
	23d	Discuss implications of the results for practice, policy, and future research.	Page 27
**OTHER INFORMATION**			
Registration and protocol	24a	Provide registration information for the review, including register name and registration number, or state that the review was not registered.	As this is a review of vaccination guidance documents, this protocol was not eligible for inclusion in PROSPERO
	24b	Indicate where the review protocol can be accessed, or state that a protocol was not prepared.	Access through requesting from corresponding authors
	24c	Describe and explain any amendments to information provided at registration or in the protocol.	Not applicable
Support	25	Describe sources of financial or non-financial support for the review, and the role of the funders or sponsors in the review.	Page 28

Competing interests	26	Declare any competing interests of review authors.	Page 29
Availability of data, code and other materials	27	Report which of the following are publicly available and where they can be found: template data collection forms; data extracted from included studies; data used for all analyses; analytic code; any other materials used in the review.	Supplementary file – all available publicly

Table S2. Search Strategies

List of Organisational Websites Reviewed

Organisation	Website
American Refugee Committee	https://wearealight.org/
AVSI Foundation	https://www.avsi.org/en
Care International UK	https://www.careinternational.org.uk/
Centre for Research on the Epidemiology of Disasters	https://www.cred.be/
Clinton Health Access Initiative	https://www.clintonhealthaccess.org/
Columbia University Mailman School of Public Health	https://www.publichealth.columbia.edu/
Concern Worldwide	https://www.concern.org.uk/
CORE Response	https://www.coreresponse.org/
Danish Refugee Council (DRC)	https://drc.ngo/
Department for International Development – Government of the United Kingdom	https://www.gov.uk/government/organisations/department-for-international-development
Department of State/Bureau of Population, Refugees, and Migration – United States	https://www.state.gov/bureaus-offices/under-secretary-for-civilian-security-democracy-and-human-rights/bureau-of-population-refugees-and-migration/
Emergency and Relief Agency – Arab Medical Union	https://darpe.me/implement-entries/arab-medical-union-emergency-relief-agency/
European Commission – Humanitarian Aid and Civil Protection	https://civil-protection-humanitarian-aid.ec.europa.eu/index_en
FHI360	https://www.fhi360.org/
Foreign Commonwealth Development Office – United Kingdom	https://www.gov.uk/government/organisations/foreign-commonwealth-development-office
GAVI	https://www.gavi.org/
Global Fund	https://www.theglobalfund.org/en/
Global Polio Eradication Initiative	https://polioeradication.org/
GOAL	https://www.goalglobal.org/
Helen Keller International	https://www.hki.org/
Hope Worldwide	https://www.hopeworldwide.org.uk/
Humanitarian Response	https://www.humanitarianresponse.info/
IMMAP	https://immap.org/
Institut Pasteur	https://www.pasteur.fr/en
InterAction	https://www.interaction.org/
International Centre for Migration and Health	https://icmhd.ch/
International Council of Nurses	https://www.icn.ch/
International Council of Voluntary Agencies	https://www.icvanetwork.org/

International Federation of the Red Cross (IFRC)	https://www.ifrc.org/
International Medical Corps	https://internationalmedicalcorps.org/
International Organization for Migration (IOM)	https://www.iom.int/
International Red Cross and Red Crescent (ICRC)	https://www.icrc.org/en/who-we-are/movement
International Rescue Committee (IRC)	https://www.rescue.org/uk
INTERSOS	https://www.intersos.org/en/
Ipas	https://www.ipas.org/
Jhpiego	https://www.jhpiego.org/
John Hopkins University for Refugee & Disaster Response	http://hopkinshumanitarianhealth.org/
John Snow, Inc.	https://www.jsi.com/
London school of Hygiene and Tropical Medicine (LSHTM)	https://www.lshtm.ac.uk/
Malaysian Medical Relief Society	https://www.mercy.org.my/
Malteser International	https://www.malteser-international.org/en.html
Medair	https://www.medair.org/
Medical Teams International	https://www.medicalteams.org/
Médecins du Monde	https://www.medecinsdumonde.org/
Médecins Sans Frontières (MSF)	https://www.msf.org/
Norwegian Refugee Council (NRC)	https://www.nrc.no/
Office of Foreign Disaster Assistance – United States of America	https://www.usaid.gov/who-we-are/organization/bureaus/bureau-democracy-conflict-and-humanitarian-assistance/office-us
Oxfam	https://www.oxfam.org.uk/
Plan International	https://plan-international.org/
Population Action International	https://pai.org/
Premiere Urgence International	https://www.premiere-urgence.org/en/
Public Health Agency of Canada	https://www.phac-aspc.gc.ca/
Public Health England	https://www.gov.uk/government/organisations/public-health-england
RedR UK	https://www.redr.org.uk/
Relief International	https://www.ri.org/
Relief Web	https://reliefweb.int/
Research for Health in Humanitarian Crises – Elrha	https://www.elrha.org/
Samaritan's Purse – International Relief	https://www.samaritanspurse.org/
Save the Children UK	https://www.savethechildren.org.uk/
Save the Children USA	https://www.savethechildren.org/
Solidarités International	https://www.solidarites.org/en/
Sphere Project	https://spherestandards.org/
Technical Network for Strengthening Immunisation	https://www.technet-21.org/en/
Terre des Hommes	https://www.terredeshommes.org/

The Harvard Humanitarian Initiative	https://hhi.harvard.edu/
UK-Med	https://www.uk-med.org/
United National Children's Fund (UNICEF)	https://www.unicef.org/
United Nations Development Programme (UNDP)	https://www.undp.org/
United Nations High Commissioner for Refugees (UNHCR)	https://www.unhcr.org/uk/
United Nations Office for the Coordination of Humanitarian Affairs (UNOCHA)	https://www.unocha.org/
United States Agency for International Development (USAID)	https://www.usaid.gov/
US Centres for Disease Control and Prevention (CDC)	https://www.cdc.gov/
Valid International	https://www.validinternational.org/
Women's Refugee Commission	https://www.womensrefugeecommission.org/
World Association for Disaster and Emergency Medicine	https://wadem.org/
World Health Organization (WHO)	https://www.who.int/
World Vision	https://www.worldvision.org.uk/

Survey Questions and Channels of Dissemination - English

<table>
<tr><td colspan="2">English Survey</td></tr>
<tr><td colspan="2">Promotional Statement[1]: If you have previously worked on vaccination in humanitarian settings and/or are aware of any vaccination guidance documents used in humanitarian settings please consider filling out this brief questionnaire for the RAISE project.

[1]Promotional statement was shared with google forms survey link to survey sharing channels</td></tr>
<tr><td colspan="2">Survey Questions:</td></tr>
<tr><td colspan="2">1. By consenting, you agree to share guidance documents with researchers for the purpose of this literature review. Participation in this survey is voluntary and does not require collection of identifiable personal information. (Required)</td></tr>
<tr><td colspan="2">2. Which organisation/agency are you currently working for? (Optional)</td></tr>
<tr><td colspan="2">3. Which country is your organisation/agency based in? (Optional)</td></tr>
<tr><td colspan="2">4. Option 1: Please share any vaccination guidance you use in humanitarian crises settings. Copy and paste the link to the document(s) below.</td></tr>
<tr><td colspan="2">5. Option 2: Please share any vaccination guidance you use in humanitarian crises settings. Upload the document(s) below.</td></tr>
<tr><td>Survey Sharing Channels:</td><td>Date Shared:</td></tr>
</table>

London School of Hygiene and Tropical Medicine Vaccine Centre Twitter	July 15, 2022
SyRG "Syria Research Group" Twitter	July 20, 2022
London School of Hygiene and Tropical Medicine Health in Humanitarian Crises Email Listserv	July 26, 2022
London School of Hygiene and Tropical Medicine Health in Humanitarian Crises Centre Twitter	July 26, 2022
SyRG "Syria Research Group" LinkedIn	July 26, 2022
SIDRA Institute Twitter	July 27, 2022
Columbia University Vaccine Safety and Confidence Working Group Email Network	August 9, 2022

Survey Questions and Channels of Dissemination - French

**French Survey**	
**Promotional Statement**[1]: Ce questionnaire et les documents de guide partagés seront utilisés pour informer une revue de la littérature sur les documents de guide pour vaccination en situations d'urgence humanitaire. Cette recherche est importante pour évaluer les directives disponibles pour vaccination en situations d'urgence humanitaire.<br><br>[1]Promotional statement was shared with google forms survey link to survey sharing channels	
**Survey Questions :**	
1. En consentant, vous nous permettez d'utiliser les documents de guides pour la revue de la littérature. Participation dans le questionnaire est volontaire et ne nécessite pas la collection d'informations personnelles identifiables. (Requis)	
2. Pour quelle organisation/agence travaillez-vous? (Optionnel)	
3. Dans quel pays votre organisation/agence est-elle située? (Optionnel)	
4. Option 1: Veuillez partager les documents de guides pour vaccination que vous utilisez dans les situations d'urgence humanitaire. **Copier et coller le(s) lien(s) dans l'espace ci-dessous.**	
5. Option 2: Veuillez partager les documents de guides pour vaccination que vous utilisez dans les situations d'urgence humanitaire. **Joindre le(s) document(s) ci-dessous.**	
**Survey Sharing Channels:**	**Date Shared:**
London School of Hygiene and Tropical Medicine Health in Humanitarian Crises Email Listserv	July 26, 2022

London School of Hygiene and Tropical Medicine Health in Humanitarian Crises Centre Twitter	July 26, 2022

Search Strategy for Guidance Documents using Google Search

**Search Equations (English and French)**	**Search Details**
(immunization OR immunisation OR vaccination OR vaccine OR vaccines) AND (guidelines OR guide OR manual OR protocol OR report OR guidance OR recommendations OR considerations or standards OR "best practices") AND ("Vaccine Preventable Diseases" OR VPD) AND ("humanitarian setting" OR "humanitarian emergencies" OR "humanitarian crises" OR "conflict zones" OR "conflict settings" OR "disaster settings")	Date of search performed: 21/07/22<br><br>Results[1]: 34,200<br><br>Number of results screened: 200
(immunisation OU immunisations OU vaccination OU vaccin OU « piqûre de rappel ») ET (directive OU « lignes directrices » OU manuel OU protocole OU rapport OU guide OU considérations OU recommandations OU normes) ET (« maladies évitables par la vaccination ») ET (« situation d'urgence humanitaire » OU « environnement humanitaire » OU « camp de réfugié » OU « catastrophe humanitaire » OU « crise humanitaire » OU « zone de conflit » OU « catastrophe naturelle »)	Date of search performed: 22/07/22<br><br>Results[1]: 6,080<br><br>Number of results screened: 200

[1]Search results were limited to dates after January 1st, 2000

Search Strategy for Guidance Documents using Bing

**Search Equations (English and French)**	**Search Details**
(immunization OR immunisation OR vaccination OR vaccine OR vaccines) AND (guidelines OR guide OR manual OR protocol OR report OR guidance OR recommendations OR considerations or standards OR "best practices") AND ("Vaccine Preventable Diseases" OR VPD) AND ("humanitarian setting" OR "humanitarian emergencies" OR "humanitarian crises" OR "conflict zones" OR "conflict settings" OR "disaster settings")	Date of search performed: 25/07/22<br><br>Results: 73,100<br><br>Number of results screened: 200
(immunisation OR immunisations OR vaccination OR vaccin) AND (directive OR "lignes directrices" OR manuel OR protocole OR rapport OR guide OR considérations OR recommandations OR normes) AND ("maladies évitables par la vaccination") AND ("situation d'urgence humanitaire" OR "environnement humanitaire" OR "catastrophe humanitaire" OR "crise humanitaire" OR "zone de conflit" OR "catastrophe naturelle")	Date of search performed: 26/07/22<br><br>Results: 3,180

	Number of results screened: 200

Search Strategies for Selected Databases[1]

[1]Separate searches conducted to include unique database subject heading terms

Global Health Database:

Topic	#	Search Equations	Results yielded (16/08/22)
Vaccine	1	(vaccin* or immuni#ation).mp. [mp=abstract, title, original title, heading words, cabicodes words]	204880
	2	exp diphtheria pertussis tetanus vaccines/ or exp diphtheria tetanus pertussis poliomyelitis vaccines/ or exp Haemophilus influenzae vaccines/ or exp poliomyelitis vaccines/ or exp measles mumps rubella vaccines/ or exp pertussis vaccines/ or vaccines/	96579
	3	1 OR 2	204880
Guidance	4	(guidance or guide* or guideline* or (instruction* or recommendation* or consideration* or best practice* or standard*) or (manual or report or protocol or document)).mp. [mp=abstract, title, original title, heading words, cabicodes words]	834337
	5	exp guidelines/	65784
	6	4 OR 5	834337
Vaccine Preventable Disease	7	(vaccine preventable disease* or VPD or (chickenpox or varicella) or diphtheria or (hepatitis A or hep A) or (hepatitis B or hep B) or (HPV or human papilloma virus) or measles or mumps or polio or pneumococcal or rotavirus or rubella or tetanus or (pertussis or whooping cough) or meningococcal or yellow fever or cholera or haemophilus influenzae type b or Hib or tuberculosis or TB).mp. [mp=abstract, title, original title, heading words, cabicodes words]	268268
	8	(Rubella virus or Mumps virus).od. or measles.sh. or mumps.sh. or rubella.sh. or pertussis.sh. or varicella.sh.	29661
	9	7 OR 8	268268
Humanitarian Emergency	10	((refugee* adj3 (camp* or settlement*)) or (humanitarian adj3 (emergenc* or crises* or setting* or area* or region* or zone*)) or (conflict adj3 (zone* or setting* or region* or emergenc*)) or (disaster adj3 (zone* or setting* or region*)) or mass displacement or food crises).mp. [mp=abstract, title, original title, heading words, cabicodes words]	2756
	11	(emergencies or conflict or natural disasters).sh.	11163

	12	10 OR 11	13325
	13	3 and 6 and 9 and 12	89
	14	Limit 13 to yr =”2000 – Current”	69

Embase Database:

Topic	#	Search Equation	Results yielded (16/08/22)
Vaccine	1	(vaccin* or immuni#ation).mp. [mp=title, abstract, heading word, drug trade name, original title, device manufacturer, drug manufacturer, device trade name, keyword heading word, floating subheading word, candidate term word]	705945
	2	Meningococcus vaccine/ or yellow fever vaccine/ or varicella zoster vaccine/ or diphtheria vaccine/ or hepatitis A hepatitis B vaccine/ or diphtheria pertussis tetanus hepatitis B vaccine/ or mumps vaccine/ or Haemophilus influenzae vaccine/ or poliomyelitis vaccine/ or measles mumps rubella vaccine/ or Rotavirus vaccine/ or diphtheria poliomyelitis tetanus vaccine/ or hepatitis A vaccine/ or diphtheria pertussis poliomyelitis tetanus hepatitis B vaccine/ or Haemophilus influenzae type b vaccine plus tetanus toxoid/ or hepatitis B vaccine/ or Haemophilus influenzae type b Meningococcus tetanus vaccine/ or cholera vaccine/ or measles vaccine/ or oral poliomyelitis vaccine/ or vaccine/ or Human papilloma virus vaccine/ or chickenpox vaccine/ or diphtheria pertussis poliomyelitis tetanus Haemophilus influenzae type b hepatitis B vaccine/ or meningitis vaccine/ or diphtheria pertussis poliomyelitis tetanus vaccine/ or mumps rubella vaccine/ or diphtheria pertussis poliomyelitis tetanus Haemophilus influenzae type b vaccine/ or diphtheria pertussis tetanus Haemophilus influenzae type b hepatitis B vaccine/ or chickenpox measles mumps rubella vaccine/ or Haemophilus influenzae type b hepatitis B vaccine/ or Haemophilus influenzae type b vaccine/ or diphtheria pertussis tetanus vaccine/ or Pneumococcus vaccine/ or diphtheria pertussis tetanus Haemophilus influenzae type b vaccine/ or BCG vaccine/ or diphtheria tetanus vaccine/ or pertussis vaccine/ or measles mumps vaccine/	215122
	3	1 OR 2	705945
Guidance	4	(guidance or guide* or guideline* or (instruction* or recommendation* or consideration* or best practice* or standard*) or (manual or report or protocol or document)).mp. [mp=title, abstract, heading word, drug trade name, original title, device manufacturer,	9439741

		drug manufacturer, device trade name, keyword heading word, floating subheading word, candidate term word]	
	5	exp practice guideline/	656783
	6	4 OR 5	9461849
	7	(vaccine preventable disease* or VPD or (chickenpox or varicella) or diphtheria or (hepatitis A or hep A) or (hepatitis B or hep B) or (HPV or human papilloma virus) or measles or mumps or polio or pneumococcal or rotavirus or rubella or tetanus or (pertussis or whooping cough) or meningococcal or yellow fever or cholera or haemophilus influenzae type b or Hib or tuberculosis or TB).mp. [mp=title, abstract, heading word, drug trade name, original title, device manufacturer, drug manufacturer, device trade name, keyword heading word, floating subheading word, candidate term word]	934908
	8	hepatitis B/ or mumps/ or rubella/ or vaccine preventable disease/ or measles/ or diphtheria/	152043
	9	7 OR 8	934908
Humanitarian Emergency	10	((refugee* adj3 (camp* or settlement*)) or (humanitarian adj3 (emergenc* or crises* or setting* or area* or region* or zone*)) or (conflict adj3 (zone* or setting* or region* or emergenc*)) or (disaster adj3 (zone* or setting* or region*)) or mass displacement or food crises).mp. [mp=title, abstract, heading word, drug trade name, original title, device manufacturer, drug manufacturer, device trade name, keyword heading word, floating subheading word, candidate term word]	5815
	11	exp humanitarian crisis/	240
	12	emergency/ or disaster/	86691
	13	10 OR 11 OR 12	91883
	14	3 and 6 and 9 and 13	163
	15	Limit 14 to yr="2000 – Current"	144

Table S3. List of included studies in the Systematic Review

Search Engines	
S1	International Federation of Red Cross and Red Crescent Societies & Johns Hopkins Bloomberg School of Public Health. The Johns Hopkins and Red Cross Red Crescent Public Health Guide in Emergencies. 2008. https://www.rcrc-resilience-southeastasia.org/wp-content/uploads/2016/09/Public-Health-Guide-in-Emergency-2nd-ed.pdf (accessed Jul 24, 2022)
S2	World Health Organization. Vaccination in Acute Humanitarian Emergencies: A Framework for Decision Making. 2017. https://apps.who.int/iris/handle/10665/255575 (accessed Aug 15, 2022)
S3	World Health Organization. Vaccination in Humanitarian Emergencies: Implementation Guide. 2017. https://www.who.int/publications/i/item/WHO-IVB-17.13 (accessed Jul 24, 2022)
S4	World Health Organization. Leave no one behind- guidance for planning and implementing catch-up vaccination. 2021. https://www.who.int/publications/i/item/9789240016514 (accessed Jul 24, 2022)
S5	World Health Organization. Delivery of immunization services for refugees and migrants. 2019. https://apps.who.int/iris/handle/10665/326924 (accessed Jul 24, 2022)
S6	World Health Organization. Communicable diseases following natural disasters: Risk assessment and priority interventions. 2006. https://www.who.int/publications/i/item/communicable-diseases-following-natural-disasters (accessed Jul 24, 2022)
S7	Checchi, F.; Freeman, R.; Mills, E. J. Public health in crisis affected populations: A practical guide for decision-makers. 2007. https://odihpn.org/wp-content/uploads/2008/05/networkpaper061.pdf (accessed May 22, 2023)
S8	World Health Organization. Planning and Implementing High-Quality Supplementary Immunization Activities for Injectable Vaccines Using an Example of Measles and Rubella Vaccines. 2016. https://www.who.int/publications/i/item/9789241511254 (accessed Jul 25, 2022)
S9	World Health Organization. Coverage and Equity: Immunisation Agenda 2030. 2021. https://www.immunizationagenda2030.org/images/documents/BLS20116_IA_Global_strategy_document_SP_3_001.pdf (accessed Jul 24, 2022)
S10	Social Science in Humanitarian Action Platform. Key Considerations: Drivers Influencing Vaccination-Related Behaviours Among Ukrainian Refugees in Poland. 2022. https://www.socialscienceinaction.org/resources/key-considerations-drivers-influencing-vaccination-related-behaviours-among-ukrainian-refugees-in-poland/ (accessed Jul 24, 2022)
S11	World Health Organization. Immunization as an essential health service- guiding principles for immunization activities during the COVID-19 pandemic and other times of severe disruption. 2020. https://www.who.int/publications/i/item/immunization-as-an-essential-health-service-guiding-principles-for-immunization-activities-during-the-covid-19-pandemic-and-other-times-of-severe-disruption (accessed Jul 25, 2022)
S12	Pan American Health Organization. Immunization Newsletter: Reducing the Risk of Vaccine-Preventable Diseases in Humanitarian Emergencies. 2021. https://iris.paho.org/handle/10665.2/55061 (accessed Jul 24, 2022)
S13	World Health Organization. Global Routine Immunization Strategies and Practices (GRISP). 2016. https://apps.who.int/iris/bitstream/handle/10665/204500/9789241510103_eng.pdf (accessed Jul 25, 2022)

S14	United Nations Children's Fund. Lessons Learned and Good Practices - Country Specific Case Studies on Immunization During the Covid-19 Pandemic. 2021. https://www.unicef.org/documents/lessons-learned-and-good-practices-country-specific-case-studies-immunization-activities (accessed Jul 24, 2022)
S15	World Health Organization. Regional Strategic Framework for Vaccine-Preventable Diseases and Immunization in the Western Pacific 2021–2030. 2022. https://apps.who.int/iris/handle/10665/359540 (accessed Jul 24, 2022)
S16	National Association of Science, Engineering, & Medicine. Improving Access and Closing the Global Immunization Gap: The Critical Public Health Value of Vaccines: Tackling Issues of Access and Hesitancy: Proceedings of a Workshop 2021. https://nap.nationalacademies.org/catalog/26134/the-critical-public-health-value-of-vaccines-tackling-issues-of (accessed Jul 25, 2022)
S17	Global Polio Eradicatioh Initiative. Reducing risk of poliomyelitis outbreaks in emergencies. 2021. https://polioeradication.org/wp-content/uploads/2021/08/20210831_Polio_in_Emergencies.pdf (accessed Aug 22, 2022)
S18	World Health Organization. Polio Endgame Strategy 2019–2023: eradication, integration, certification and containment. 2019. https://apps.who.int/iris/handle/10665/329948 (accessed Jul 24, 2022)
S19	Inter-Agency Working Group on Reproductive Health in Crises. La santé du nouveau-né en situations de crise humanitaire: Guide de Terrain. 2017. https://www.healthynewbornnetwork.org/hnn-content/uploads/French-Newborn-Health-in-Humanitarian-Settings-Field-Guide-1-1.pdf (accessed Jul 27, 2022)
S20	World Health Organization. Procédures opérationnelles stanndardisées: riposte à un évènement ou à une flambée de poliomyélite. 2020. https://www.who.int/fr/publications-detail/9789240002999 (accessed Jul 27, 2022)
Organisational Website Searches	
S21	World Health Organization. Ensuring the integration of refugees and migrants in immunization policies, planning and service delivery globally. 2022. https://www.who.int/publications/i/item/9789240051843 (accessed Jul 24, 2022)
S22	World Health Organization. Manual for the health care of children in humanitarian emergencies. 2008. https://www.who.int/publications/i/item/9789241596879 (accessed Jul 24, 2022)
S23	World Health Organization. Regional Office for Europe - Guidance on vaccination and prevention of vaccine preventable disease outbreaks for countries hosting refugees from Ukraine. 2022. https://apps.who.int/iris/handle/10665/353408 (accessed Jul 24, 2022)
S24	International Committee of the Red Cross. Nursing Guidelines. 2021. https://shop.icrc.org/the-icrc-guidelines-for-teaching-nursing-care-and-icrc-nursing-guidelines-working-with-limited-resources-in-armed-conflict-and-other-situations-of-violence-pdf-en.html (accessed Jul 24, 2022)
S25	Médecins Sans Frontières. Management of a Measles Epidemic. 2013. https://medicalguidelines.msf.org/en/viewport/mme/english/management-of-a-measles-epidemic-30542833.html (accessed Jul 24, 2022)
S26	Sphere Association. The Sphere Handbook: Humanitarian Charter and Minimum Standards in Humanitarian Response. 2018. https://spherestandards.org/wp-content/uploads/Sphere-Handbook-2018-EN.pdf (accessed Jul 25, 2022)
S27	Yale Institute for Global Health & United Nations Children's Fund. Vaccine Misinformation Management Field Guide. 2020. https://www.unicef.org/mena/reports/vaccine-misinformation-management-field-guide (accessed Jul 24, 2022)

S28	Save the Children. Not Immune - Children in Conflict. 2020. https://resourcecentre.savethechildren.net/document/not-immune-children-conflict/ (accessed Jul 24, 2022)
S29	United Nations Human Rights Council. Handbook for Emergencies - Fourth Edition. 2018. Available online: https://emergency.unhcr.org/ (accessed Jul 28, 2022)
Peer Reviewed Databases	
S30	Jalloh MF, Wilhelm E, Abad N, Prybylski D. Mobilize to vaccinate: lessons learned from social mobilization for immunization in low and middle-income countries. Human Vaccines and Immunotherapeutics. 2020;16(5):1208-14.
S31	Deen J, Seidlein Lv, Luquero FJ, Troeger C, Reyburn R, Lopez AL, et al. The scenario approach for countries considering the addition of oral cholera vaccination in cholera preparedness and control plans. Lancet Infectious Diseases. 2016;16(1):125-9.
S32	Nnadi C, Etsano A, Uba B, Ohuabunwo C, Melton M, Nganda GW, et al. Approaches to vaccination among populations in areas of conflict. Special Issue: Polio endgame & legacy-implementation, best practices, and lessons learned. 2017;216(Suppl. 1):S368-S72.
S33	Moodley K, Hardie K, Selgelid MJ, Waldman RJ, Strebel P, Rees H, et al. Ethical considerations for vaccination programmes in acute humanitarian emergencies. Bulletin of the World Health Organization. 2013;91(4):290-7.
S34	Lam E, McCarthy A, Brennan M. Vaccine-preventable diseases in humanitarian emergencies among refugee and internally-displaced populations. Human Vaccines and Immunotherapeutics. 2015;11(11):2627-36.
S35	Finkelstein P, Teisch L, Allen CJ, Ruiz G. Tetanus: A Potential Public Health Threat in Times of Disaster. Prehospital and disaster medicine. 2017;32(3):339-42.
S36	Leach K, Checchi F. The utilisation of vaccines in humanitarian crises, 2015-2019: a review of practice. Vaccine. 2022;40(21):2970-8.
Online Survey	
S37	World Health Organization. Training for mid-level managers. Module 6: Making a comprehensive annual national immunization plan and budget. 2020. https://www.who.int/publications/i/item/module-6-making-a-comprehensive-annual-national-immunization-plan-and-budget (accessed Jul 25, 2022)
S38	Republic of Sudan. Comprehensive Multi-Year National Immunization Plan 2006-2010. 2005. http://www.fmoh.gov.sd/St_Paln/MYP2006-2010.pdf (accessed Aug 12, 2022)
S39	Médecins Sans Frontières. Management of a Cholera Epidemic. 2018. https://samumsf.org/sites/default/files/2018-10/Management%20of%20a%20Cholera%20Epidemic.pdf (accessed Jul 25, 2022)
Reviewing reference lists of included documents	
S40	United Nations Children's Fund. Emergency Field Handbook. 2005. https://www.humanitarianlibrary.org/resource/emergency-field-handbook-guide-unicef-staff-0 (accessed Aug 25, 2022)
S41	World Health Organization. Oral cholera vaccine in mass immunization campaigns. 2010. https://apps.who.int/iris/handle/10665/44448 (accessed Aug 25, 2022)
S42	United Nations Refugee Agency. Operational guidelines on improving newborn health in refugee operations. 2013. https://www.unhcr.org/protection/health/54bd0dc49/operational-guidelines-improving-newborn-health-refugee-operations.html (accessed Aug 25, 2022)
S43	United States Institute of Peace. Defying expectations: polio vaccination programs amid political and armed conflict. 2010. https://www.usip.org/sites/default/files/PB%2064%20-%20Polio%20Vaccination%20Programs%20Amid%20Political%20and%20Armed%20Conflict.pdf (accessed Aug 22, 2022)
S44	World Health Organization, United Nations Refugee Agency, United Nations Children's Fund. Joint statement on general principles on vaccination of refugees, asylum-seekers, and

	migrants in the WHO European Region. 2015. https://apps.who.int/iris/handle/10665/362391 (accessed Aug 22, 2022)
S45	World Health Organization. Guiding principles for recovering, building resiliency, and strengthening of immunization in 2022 and beyond. 2022. https://www.linkedimmunisation.org/resources/guiding-principles-for-recovering-building-resiliency-and-strengthening-of-immunisation-in-2022-and-beyond/ (accessed Aug 25, 2022)
S46	United Nations Children's Fund. Reducing Measles Mortality in Emergencies. 2004. https://apps.who.int/iris/handle/10665/68744 (accessed Aug 22, 2022)
S47	World Health Organization. Communicable Disease Control in Emergencies. 2005. https://apps.who.int/iris/handle/10665/96340 (accessed Aug 22, 2022)
Reviewing the reference list of Aboubaker et al.	
S48	Inter-Agency Working Group on Reproductive Health in Crises. Field Manual on Reproductive Health in Humanitarian Settings. 2018. https://iawgfieldmanual.com/manual (accessed Jul 25, 2022)

Table S4. Data extraction classification definitions

Crisis typologies and contexts included[1]
1. Sudden mass displacement: a large proportion of the population moving away from the community of habitual residence.<br>2. Armed conflict: direct exposure of the civilian, non-combatant population to episodes of armed conflict.<br>3. Food crises: deterioration of nutritional status beyond and above seasonal fluctuations and situations of chronic poor nutritional status and/or food insecurity.<br>4. Natural or industrial disaster: increased risk of exposure to adverse weather conditions, famine, drought, environmental degradation for a large proportion of the population.<br>5. Complex humanitarian emergency: breakdown of critical administrative and management functions within public and/or private sector, caused by a complicated set of social, health, economic, and political circumstances leading to disruption of public health and related services.<br>6. Refugee camps: temporary settlement built to receive people who have been displaced from their place of habitual residence in response to crisis<br>7. Epidemics: elevation of death rate in response to a confirmed infectious disease.<br><br>[1] Crises typology definitions adapted from WHO's Vaccination in Acute Humanitarian Emergencies: A Framework for Decision Making
Functional classification of guidance
1. Technical Normative Guidance: defined as guidance that provides detailed information on VPD population risk factors and what to do regarding vaccination interventions. It can contain standards of care and recommendations on what vaccines to administer, age ranges, and dosing. It also includes derivative products like for example a routine vaccination schedule.<br>2. Operational Guidance: defined as guidance that describes in detail how to implement interventions recommended in the technical normative guidance. It includes operational manuals, tool kits, handbooks, etc.<br>3. Descriptive Guidance: defined as guidance that provides general information but does not include in detail what to do. This includes fact sheets, frameworks, or policy documents.<br>4. Ethical Guidance: defined as normative guidance based on principles or conventions. The guidance may not be based on hard science or evidence but rather on a moral philosophical framework.<br>5. Evidence Reviews: defined as a synthesis of evidence or narrative review related to a specific aspect of vaccination. Guidance is typically given as a summary of findings.
Documents were also classified based on the crisis phases or scenarios they focussed on:
1. Acute Crisis: a sudden unplanned displacement of a large proportion of the population, direct exposure of the civilian population to new or exacerbated and sustained episodes of armed conflict, impending or already occurred sudden deterioration of nutritional status, natural or industrial disasters such as floods, earthquakes, or tsunamis, and/or sudden breakdown of critical administrative and management functions which result in large-scale disruption of public health and related services<br>2. Recovery: activities to catch up on low coverage following a disruption to routine vaccination services, typically following an acute crisis.<br>3. Host countries: focused on integrating refugees into national immunization programs.

Table S5. Data extraction Framework

Dimension	Categories	Sub-categories
Guidance Identification	Title	
	Lead Author/Organisation	
	Publication Date	
	URL	
	Guidance Type	Technical<br>Operational<br>Descriptive<br>Ethical<br>Evidence Review
Context	Intended Target User(s) of Guidance	National or Subnational Immunisation Programme Planners or Managers<br>INGOs<br>Medical Professional<br>Policy Makers<br>Advocacy or Communication Organisations or Professionals<br>Field Workers<br>Journalists<br>Students<br>Government Officials
	Intended Response Focus of Guidance	Acute Crises<br>Recovery<br>Host Country
	Intended Beneficiary Groups Identified	Neonate<br>Infant<br>Children<br>Adolescent<br>Pregnant Women<br>Adult
	Type of Humanitarian Crises	Armed Conflict<br>Complex Humanitarian Emergency<br>Epidemic<br>Food Crises<br>Humanitarian Emergencies - Unspecified<br>Natural or Industrial Disaster<br>Refugee Camp<br>Sudden Unplanned Displacement
	Identification of the term 'Zero-Dose' or 'Zero-Dose Children'	Yes<br>No
Vaccine Focus	Type of Vaccine Preventable Disease Guidance	Cholera, Diphtheria, Hep A, Hep B, Hib, HPV, Measles, Meningococcal, Mumps, Pertussis, Polio, Pneumococcal, Rotavirus, Rubella, Tetanus, Tuberculosis, Varicella, Yellow Fever
Dosing Guidance (if available)	Dose	
	Target Age Group	
	Modality of Delivery	Mass and Routine<br>Mass<br>Routine<br>Unspecified Modality

Table S6. Vaccine preventable diseases included in guidance documents

**Legend:** X = guidance on VPD included in document		**Vaccine Preventable Diseases included in guidance**																		
		**WHO Vaccines recommended for all routine immunization programs**												**WHO Non-universal vaccines recommended for routine immunization programs**						Number of VPDs by document n/18 (%)
**Guidance document**		Diphtheria	Tetanus	Pertussis	Hep B	Hib	HPV	Measles	Polio	Pneumo-coccal	Rotavirus	Rubella	TB	Varicella	Mumps	Cholera	Hep A	Meningo-coccal	Yellow Fever	
1	Vaccination in Acute Humanitarian Emergencies: A Framework for Decision Making	x	x	x	x	x	x	x	x	x	x	x	x	x	x	x	x	x	x	18 (100)
2	Leave No One Behind – Guidance for Planning and Implementing Catch-Up Vaccination	x	x	x	x	x	x	x	x	x	x	x	x	x	x	x	x	x	x	18 (100)
3	Delivery of Immunization Services for Refugees and Migrants – Technical Guidance	x	x	x	x	x	x	x	x	x		x	x	x	x			x	x	15 (83)
4	Communicable Diseases Following Natural Disasters: Risk Assessment and Priority Interventions		x					x								x	x			4 (22)
5	Public Health in Crises-Affected Populations: A Practical Guide for Decision-Makers	x	x	x	x	x		x	x	x	x	x	x		x	x	x		x	15 (83)
6	Public Health Guide for Emergencies – Second Edition	x	x	x	x			x	x				x			x	x	x	x	11 (61)
7	Ensuring the integration of Refugees and Migrants in Immunization Policies, Planning, and Service Delivery Globally	x	x	x	x	x	x	x	x	x	x	x	x	x	x	x	x	x	x	18 (100)
8	Manual for the Healthcare of Children in Humanitarian Emergencies	x	x	x	x	x		x	x	x			x		x			x	x	12 (67)
9	Guidance on Vaccination and Prevention of Vaccine Preventable Disease Outbreaks for Countries Hosting Refugees from Ukraine	x	x	x	x	x		x	x	x	x	x	x	x				x		13 (72)
10	ICRC Nursing Guidelines		x		x			x			x					x				5 (28)
11	Management of a Measles Epidemic							x												1 (6)
12	Management of a Cholera Epidemic															x				1 (6)
13	Comprehensive Multi-Year Immunization Plan 2006-2010	x	x	x	x	x		x	x	x	x	x	x					x	x	13 (72)
14	UNHCR Emergency Handbook	x	x	x	x	x		x	x				x							8 (44)
15	La Santé du Nouveau-Née en Situations de Crise Humanitaire		x		x				x	x			x			x		x		7 (39)
16	Procédures Opérationnelles Standardisées Riposte à un Évènement ou à une Flambé de Poliomyélite								x											1 (6)
17	The Sphere Handbook: Humanitarian Charter and Minimum Standards in Humanitarian Response	x	x	x	x			x	x		x	x			x	x	x		x	12 (67)
18	Inter-agency field manual on reproductive health in humanitarian settings		x		x		x													3 (17)
19	Vaccination in Acute Humanitarian Emergencies: Implementation Guide	x	x	x	x	x	x	x	x	x	x	x	x	x	x	x	x	x	x	18 (100)
20	Making a Comprehensive Annual National Immunization	x	x	x	x			x	x			x	x		x				x	10 (56)
21	Vaccine Misinformation Field Guide						x	x	x											3 (17)
22	Not Immune: Children in Conflict	x	x	x		x		x	x	x	x					x			x	10 (56)
23	Planning and Implementing High-Quality Supplementary Immunization Activities for Injectable Vaccines Using an Example of Measles and Rubella Vaccines	x	x	x	x	x		x	x	x		x	x	x	x				x	13 (72)

	24	Immunization Agenda 2030: Coverage and Equity		x				x	x	x			x				x				6 (33)
	25	Key Considerations: Drivers Influencing Vaccination Related Behaviour Among Ukrainian Refugees in Poland	x	x	x	x	x		x	x			x	x	x	x					11 (61)
	26	Immunization as an Essential Health Service: Guiding Principles for Immunization Activities during the COVID-19 Pandemic and other Times of Severe Disruption				x		x	x												3 (17)
	27	Reducing the Risk of Vaccine-Preventable Diseases in Humanitarian Emergencies							x	x			x								3 (17)
	28	Global Routine Immunization on Strategies and Practices	x	x	x	x		x	x	x	x	x	x	x			x		x		13 (72)
	29	Polio Endgame Strategy 2019-2023 : Eradication, Integration, Certification and Containment								x											1 (6)
	30	Regional Strategic Framework for Vaccine-Preventable Diseases and Immunization in the Western Pacific 2021-2030	x	x	x	x	x	x	x	x	x	x	x	x	x	x	x	x	x	x	18 (100)
	31	Lessons Learned and Practices: Country-Specific Case Studies on Immunization Activities During the Covid-19 Pandemic	x	x	x	x	x		x	x	x	x	x	x			x			x	13 (72)
	32	Mobilize to Vaccinate: Lessons Learned from Social Mobilization for Immunization in Low- and Middle-Income Countries		x	x			x	x	x			x				x				7 (39)
	33	The Scenario Approach for Countries Considering the Addition of Oral Cholera Vaccination in Cholera Preparedness and Control Plans															x				1 (6)
	34	Approaches to Vaccination Among Population in Areas of Conflict								x											1 (6)
	35	Improving Access and Closing the Global Immunization Gap – The Critical Public Health Value of Tackling Issues of Access and Hesitancy	x	x	x		x		x	x							x		x	x	9 (50)
	36	Ethical Considerations for Vaccination Programmes in Acute Humanitarian Emergencies							x												1 (6)
	37	Emergency Field Handbook		x					x								x				3 (17)
	38	Oral Cholera Vaccine in Mass Immunization Campaigns															x				1 (6)
	39	Operational Guidelines on Improving Newborn Health in Refugee Operations		x		x				x											3 (17)
	40	Reducing Risk of Poliomyelitis Outbreaks in Emergencies							x	x											2 (11)
	41	Defying Expectations: Polio Vaccination Programs Amid Political and Armed Conflict								x											1 (6)
	42	Joint Statement on General Principles on Vaccination of Refugees, Asylum-Seekers, and Migrants in the WHO European Region				x			x	x			x			x					5 (28)
	43	Guiding Principles for Recovering, Building Resiliency, and Strengthening of Immunization in 2022 and Beyond							x	x						x				x	4 (22)
	44	Reducing Measles Mortality in Emergencies							x												1 (6)
	45	Communicable Disease Control in Emergencies	x	x	x	x			x	x	x			x		x	x	x	x	x	12 (67)
	46	Vaccine-Preventable Diseases in Humanitarian Emergencies Among Refugee and Internally Displaced Populations		x		x	x		x	x	x	x	x		x	x	x	x	x	x	14 (78)
	47	Tetanus: A Potential. Public Health Threat in Times of Disaster		x																	1 (6)
	48	The Utilisation of Vaccines in Humanitarian Crises 2015-2019: A Review of Practice	x	x	x	x	x	x	x		x	x	x				x				11 (61)
		Total documents by VPD n/48 (%)	23 (48)	32 (67)	23 (48)	27 (56)	18 (38)	13 (27)	37 (77)	35 (73)	18 (38)	15 (31)	21 (44)	19 (40)	10 (21)	16 (33)	24 (50)	11 (23)	15 (31)	19 (40)	

Table S7. AGREE II critical appraisal by domain

Corresponding Document in Data Extraction Table	Quality Appraisal Score by AGREE II Domain (%)					
	Scope and Purpose /21	Stakeholder Involvement /21	Rigour of Development /56	Clarity of Presentation /21	Applicability /28	Editorial Independence /21
Vaccination in Acute Humanitarian Emergencies: A Framework for Decision Making	21 (100)	16 (76)	33 (59)	18 (86)	22 (79)	8 (57)
Leave No One Behind – Guidance for Planning and Implementing Catch-Up Vaccination	20 (95)	10 (48)	21 (38)	17 (81)	17 (61)	4 (28)
Delivery of Immunization Services for Refugees and Migrants – Technical Guidance	20 (95)	15 (71)	38 (68)	16 (76)	19 (68)	6 (43)
Communicable Diseases Following Natural Disasters: Risk Assessment and Priority Interventions	15 (71)	10 (48)	22 (39)	16 (76)	18 (64)	5 (36)
Public Health in Crises-Affected Populations: A Practical Guide for Decision-Makers	21 (100)	15 (71)	22 (39)	16 (76)	21 (75)	11 (79)
Public Health Guide for Emergencies – Second Edition	21 (100)	16 (76)	29 (52)	19 (90)	22 (79)	10 (71)
Ensuring the integration of Refugees and Migrants in Immunization Policies, Planning, and Service Delivery Globally	21 (100)	13 (62)	39 (70)	16 (76)	13 (46)	10 (71)
Manual for the Healthcare of Children in Humanitarian Emergencies	20 (95)	15 (71)	28 (50)	15 (71)	19 (68)	10 (71)
Guidance on Vaccination and Prevention of Vaccine Preventable Disease Outbreaks for Countries Hosting Refugees from Ukraine	20 (95)	6 (29)	13 (23)	13 (62)	16 (57)	4 (29)
ICRC Nursing Guidelines	21 (100)	12 (57)	28 (50)	18 (86)	22 (79)	5 (36)
Management of a Measles Epidemic	19 (90)	14 (67)	21 (38)	14 (67)	21 (75)	4 (29)

Management of a Cholera Epidemic	20 (95)	12 (57)	20 (36)	15 (71)	20 (71)	6 (43)
Comprehensive Multi-Year Immunization Plan 2006-2010	16 (76)	10 (48)	19 (34)	16 (76)	15 (54)	6 (43)
UNHCR Emergency Handbook	16 (76)	11 (52)	30 (54)	18 (86)	15 (54)	5 (36)
La Santé du Nouveau-Née en Situations de Crise Humanitaire	21 (100)	13 (62)	25 (45)	15 (71)	19 (68)	6 (43)
Procédures Opérationnelles Standardisées Riposte à un Évènement ou à une Flambé de Poliomyélite	21 (100)	13 (62)	23 (41)	14 (67)	16 (57)	4 (29)
The Sphere Handbook: Humanitarian Charter and Minimum Standards in Humanitarian Response	21 (100)	16 (76)	33 (59)	18 (86)	22 (79)	9 (64)
Inter-agency field manual on reproductive health in humanitarian settings	17 (81)	10 (48)	23 (41)	13 (62)	17 (61)	6 (43)
Vaccination in Acute Humanitarian Emergencies: Implementation Guide	20 (95)	16 (76)	34 (61)	17 (81)	19 (68)	6 (43)
Making a Comprehensive Annual National Immunization	21 (100)	16 (76)	22 (39)	16 (76)	19 (68)	5 (36)
Vaccine Misinformation Field Guide	20 (95)	11 (52)	19 (34)	17 (81)	15 (54)	9 (64)
Not Immune: Children in Conflict	17 (81)	14 (67)	36 (64)	13 (62)	14 (50)	8 (57)
Planning and Implementing High-Quality Supplementary Immunization Activities for Injectable Vaccines Using an Example of Measles and Rubella Vaccines	19 (90)	13 (62)	21 (38)	15 (71)	20 (71)	6 (43)
Immunization Agenda 2030: Coverage and Equity	20 (95)	4 (19)	16 (29)	14 (67)	15 (53)	4 (29)
Key Considerations: Drivers Influencing Vaccination Related Behaviour Among Ukrainian Refugees in Poland	20 (95)	17 (81)	21 (38)	16 (76)	12 (43)	12 (86)
Immunization as an Essential Health Service: Guiding Principles for	21 (100)	8 (38)	19 (34)	15 (71)	15 (54)	11 (79)

Immunization Activities during the COVID-19 Pandemic and other Times of Severe Disruption						
Reducing the Risk of Vaccine-Preventable Diseases in Humanitarian Emergencies	9 (43)	7 (33)	12 (21)	13 (62)	6 (21)	7 (50)
Global Routine Immunization on Strategies and Practices	19 (90)	13 (62)	25 (45)	16 (76)	17 (61)	5 (36)
Polio Endgame Strategy 2019-2023 : Eradication, Integration, Certification and Containment	20 (95)	16 (76)	31 (55)	17 (81)	17 (61)	6 (43)
Regional Strategic Framework for Vaccine-Preventable Diseases and Immunization in the Western Pacific 2021-2030	18 (86)	11 (53)	23 (41)	13 (62)	16 (57)	4 (29)
Lessons Learned and Practices: Country-Specific Case Studies on Immunization Activities During the Covid-19 Pandemic	18 (86)	14 (67)	34 (61)	17 (81)	22 (79)	4 (29)
Mobilize to Vaccinate: Lessons Learned from Social Mobilization for Immunization in Low- and Middle-Income Countries	17 (81)	10 (48)	27 (48)	13 (62)	17 (61)	11 (79)
The Scenario Approach for Countries Considering the Addition of Oral Cholera Vaccination in Cholera Preparedness and Control Plans	17 (81)	10 (48)	23 (41)	15 (71)	15 (53)	5 (36)
Approaches to Vaccination Among Population in Areas of Conflict	15 (71)	12 (57)	26 (46)	13 (62)	12 (43)	11 (79)
Improving Access and Closing the Global Immunization Gap – The Critical Public Health Value of Tackling Issues of Access and Hesitancy	17 (81)	8 (38)	14 (25)	11 (52)	14 (50)	6 (43)
Ethical Considerations for Vaccination Programmes in Acute Humanitarian Emergencies	17 (81)	14 (67)	17 (30)	14 (67)	10 (36)	5 (36)
Emergency Field Handbook	17 (81)	10 (48)	15 (27)	15 (71)	22 (79)	8 (57)

Oral Cholera Vaccine in Mass Immunization Campaigns	16 (76)	12 (57)	21 (38)	15 (71)	21 (75)	5 (36)
Operational Guidelines on Improving Newborn Health in Refugee Operations	17 (81)	12 (57)	21 (38)	16 (76)	19 (68)	5 (36)
Reducing Risk of Poliomyelitis Outbreaks in Emergencies	12 (57)	8 (38)	10 (18)	11 (52)	9 (32)	3 (21)
Defying Expectations: Polio Vaccination Programs Amid Political and Armed Conflict	17 (81)	14 (67)	25 (45)	13 (62)	16 (57)	4 (29)
Joint Statement on General Principles on Vaccination of Refugees, Asylum-Seekers, and Migrants in the WHO European Region	13 (62)	8 (38)	18 (32)	10 (48)	7 (25)	5 (36)
Guiding Principles for Recovering, Building Resiliency, and Strengthening of Immunization in 2022 and Beyond	16 (76)	10 (48)	18 (32)	13 (62)	7 (25)	9 (64)
Reducing Measles Mortality in Emergencies	10 (48)	11 (52)	14 (25)	10 (48)	12 (43)	4 (29)
Communicable Disease Control in Emergencies	8 (38)	9 (43)	10 (18)	13 (62)	14 (50)	3 (21)
**SUMMARY**	**85%**	**57%**	**41%**	**71%**	**58%**	**46%**

Table S8. AGREE II – Critical Appraisal disaggregated by individual domain indicators[1]

Domain	Indicators							
**1 Scope and Purpose**	1. The overall objective of the guideline is (are) specifically described	2. The health question(s) covered by the guideline is (are) specifically described.	3. The population (patients, public, etc.) to whom the guideline is meant to apply is specifically described.					
**Average Score**	**6·3**	**5·9**	**5·6**					
**2 Stakeholder involvement**	4. The guideline development group includes individuals from all relevant professional groups.	5. The views and preferences of the target population (patients, public, etc.) have been sought	6. The target users of the guideline are clearly defined.					
**Average Score**	**3·9**	**3·1**	**4·9**					
**3 Rigour of Development**	7. Systematic methods were used to search for evidence.	8. The criteria for selecting the evidence are clearly described.	9. The strengths and limitations of the body of evidence are clearly described.	10. The methods for formulating the recommendations are clearly described.	11. The health benefits, side effects, and risks have been considered in formulating the recommendations.	12. There is an explicit link between the recommendations and the supporting evidence.	13. The guideline has been externally reviewed by experts prior to its publication	14. A procedure for updating the guideline is provided.
**Average Score**	**2·5**	**2·3**	**2·3**	**3·2**	**3·4**	**4·0**	**3·9**	**1·5**
**4 Clarity of Presentation**	15. The recommendations are specific and unambiguous	16. The different options for management of the condition or health issue are clearly presented.	17. Key recommendations are easily identifiable.					
**Average Score**	**4·7**	**4·8**	**5·4**					
**5 Applicability**	18. The guideline describes facilitators and barriers to its application.	19. The guideline provides advice and/or tools on how the recommendations can be put into practice.	20. The potential resource implications of applying the recommendations have been considered.	21. The guideline presents monitoring and/or auditing criteria.				
**Average Score**	**4·4**	**4·2**	**4·1**	**3·6**				
**6 Editorial Independence**	22. The views of the funding body have not influenced the content of the guideline.	23. Competing interests of guideline development group members have been recorded and addressed.						
**Average Score**	**4·2**	**2·4**						

[1]Average score for 45 guidelines ranked by Likert Scale: (/7) 1- Strongly Disagree 7- Strongly Agree

Table S9. SANRA – Critical Appraisal by Item

Corresponding Document in Data Extraction Table	Quality Appraisal Score by SANRA Evaluation Item					
	1 **Justification of the Article's Importance for the Readership** /2	2 **Statement of Concrete Aims or Formulation of Questions** /2	3 **Description of Literature Search** /2	4 **Referencing** /2	5 **Scientific Reasoning** /2	6 **Appropriate Presentation of Data** /2
Vaccine-Preventable Diseases in Humanitarian Emergencies Among Refugee and Internally Displaced Populations	2	2	2	2	1	1
Tetanus: A Potential. Public Health Threat in Times of Disaster	1	1	2	2	1	2
The Utilisation of Vaccines in Humanitarian Crises 2015-2019: A Review of Practice	2	1	2	2	2	2
SUMMARY	83%	67%	100%	100%	67%	83%
